# Ionic liquid-caged nucleic acids enable active folding-based molecular recognition with hydrolysis resistance

**DOI:** 10.1093/nar/gkad1093

**Published:** 2023-11-22

**Authors:** Byunghwa Kang, Soyeon V Park, Seung Soo Oh

**Affiliations:** Department of Materials Science and Engineering, Pohang University of Science and Technology (POSTECH), Pohang, Gyeongbuk 37673, South Korea; Department of Materials Science and Engineering, Pohang University of Science and Technology (POSTECH), Pohang, Gyeongbuk 37673, South Korea; Department of Materials Science and Engineering, Pohang University of Science and Technology (POSTECH), Pohang, Gyeongbuk 37673, South Korea; Institute for Convergence Research and Education in Advanced Technology (I-CREATE), Yonsei University, Incheon 21983, South Korea

## Abstract

Beyond storage and transmission of genetic information in cellular life, nucleic acids can perform diverse interesting functions, including specific target recognition and biochemical reaction acceleration; the versatile biopolymers, however, are acutely vulnerable to hydrolysis-driven degradation. Here, we demonstrate that the cage effect of choline dihydrogen phosphate permits active folding of nucleic acids like water, but prevents their phosphodiester hydrolysis unlike water. The choline-based ionic liquid not only serves as a universal inhibitor of nucleases, exceptionally extending half-lives of nucleic acids up to 6 500 000 times, but highly useful tasks of nucleic acids (e.g. mRNA detection of molecular beacons, ligand recognition of aptamers, and transesterification reaction of ribozymes) can be also conducted with well-conserved affinities and specificities. As liberated from the function loss and degradation risk, the presence of undesired and unknown nucleases does not undermine desired molecular functions of nucleic acids without hydrolysis artifacts even in nuclease cocktails and human saliva.

## Graphical Abstract

**Graphical Abstract:**
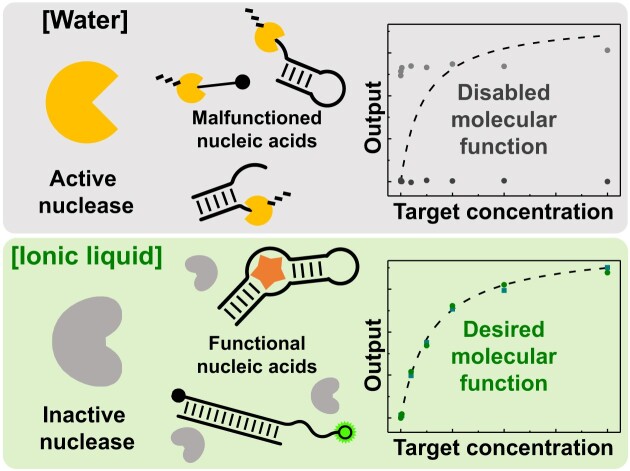

## Introduction

The capability of nucleic acids (e.g. DNAs and RNAs) is no longer limited to storage and transmission of genetic information (1). Either in or out of cells, they have proven diverse functionalities; like membrane receptors, nucleic acid-based aptamers can specifically recognize target molecules, sometimes accompanying conformational changes, and like enzymes, ribozymes and DNAzymes catalyze a variety of biochemical reactions (2–4). Since the highly useful functions arise from the unique sequence of nucleic acids and relevant folding structures, they are frequently intimidated by the hydrolytic mechanism that all kinds of organisms have evolved to provide in removing unnecessary nucleic acids (e.g. genetically damaged primers in DNA replication, defective transcripts in translation, and even viral RNAs against infection) (5–9). For example, the RNA aptamer targeting human tumor necrosis factor α (hTNF-α) is known to reduce inflammation by its hTNF-α inhibition, but it fails to withstand nucleolytic degradation, vanishing within <10 min in 85% human serum (10). Although the hammerhead ribozyme occurs in nature, the half-life of its derivatives is <10 s in a physiological condition, thereby frustrating its genetic scissor-like *in vivo* uses (11).

Against the nucleolytic degradation, there have been considerable efforts to protect functional nucleic acids (FNAs) (5). To decelerate spontaneous hydrolysis and reduce activities of nucleases, which are found everywhere in biological fluids (e.g. saliva, urine, sweat, tear and blood) (12,13), DNAs and RNAs are typically stored at low temperature (e.g. lower than –20°C) (14). However, they must be thawed prior to use and subsequently mixed with suitable buffers, perhaps leading to freeze-thaw cycles-driven degradation and nuclease contamination (15,16). Heat treatments can denature or inactivate many kinds of enzymes, but several nucleases, such as RNase A family, are known to survive even after prolonged boiling and autoclaving (17). Alternatively, to enhance nuclease resistance of FNAs, various chemical modifications (e.g. 2′-*O*-methylation, phosphorothioate substitution, incorporation of 2′-5′/3′-5′ backbone heterogeneity, and 3′-capping) have been extensively investigated (18–20), while the inclusion of artificial nucleotides does not guarantee to maintain the pre-designed functions of the nucleic acids (21–23). Despite the availability of nuclease-resistant xeno nucleic acids (24), their resistance against other enzymes (e.g. polymerases) inevitably makes their functionalization process significantly difficult (25,26). Taken together, it is extremely challenging to achieve both the protection and desired function of nucleic acids.

Interestingly, hydrogen bonding of nucleic acids can be maintained in non-canonical solvents, such as ionic liquids and organic solvents (27,28). In particular, the effectiveness of the donor-acceptor interactions (29) allows some ionic liquids to coexist with naturally occurring DNA helical structures (e.g. B-form duplex (30), G-quadruplex (31) and triplex (32)) although complex conformations of nucleic acids and their relevant molecular functions (33) have not been explored yet. In principle, the molten salts should exhibit high ionic strength that can disrupt the water cage surrounding charged molecules (34–36); several combinations of cations and anions conserved DNAs from both spontaneous and biological degradation (16,37), and importantly, choline dihydrogen phosphate (CDHP) proved its exceptional ability to reduce hydrolytic activities of two well-known nucleases (DNase I and RNase A) (38,39). From these two kinds of attractiveness, it would be reasonable to question whether a designer solvent, such as CDHP, may serve as a potent dual-role solvent that specifically inhibits hydrolytic actions of nuclease enzymes while allowing conformational and functional diversities of nucleic acids.

In this study, we demonstrate that the cage effect of CDHP readily allows active conformational folding of FNAs like water, but completely blocks their hydrolytic degradation unlike water. In the CDHP, the highly useful functions of FNAs, directly derived from their unique sequence and relevant folding structures, are as effective as in an aqueous buffer, so mRNA-dependent conformational changes of molecular beacon, aptameric target recognition events, and ribozyme-mediated phosphoryl transfer reactions were successfully observed with well-conserved affinities and specificities. Importantly, the simple CDHP cage exclusively offers a hydrolytically unfavorable situation that can inhibit both spontaneous and nuclease-driven degradation; accordingly, nucleic acids were remarkably resistant to all kinds of nucleases (e.g. RNases, DNases, endonucleases and exonucleases), recording the longest DNA and RNA half-lives (up to 6 500 000-fold larger than in optimal buffers at 37°C). Despite the presence of undesired and unknown nucleases, the CDHP-mediated nucleic acid shielding was proven to guarantee no nucleolytic degradation and function loss, achieving desired molecular functions of nucleic acids without their hydrolysis-related artifacts even in clinically important or contaminated samples, such as human saliva and nuclease cocktails.

## Materials and methods

### Reagents and materials

Oligonucleotides were synthesized by Bioneer (Daejeon, Korea) or Integrated DNA Technologies (IDT) (Coralville, IA) (Supplementary Table S1). Choline dihydrogen phosphate was purchased from IoLiTec (Heilbronn, Germany). Sodium chloride (NaCl), potassium chloride (KCl), magnesium chloride (MgCl_2_) hexahydrate, HEPES, Tris(hydroxymethyl)aminomethane (Tris) base, Tris hydrochloride and agarose were purchased from GA Biochem (Chuncheon, Korea). 100 mM ATP, UTP, GTP, CTP solutions and 10× Dulbecco phosphate buffered saline (DPBS) were purchased from Tech and Innovation (Chuncheon, Korea). Sodium hydroxide (NaOH), Thioflavin T and SYBR™ Green I Nucleic Acid Gel Stain were purchased from Sigma-Aldrich (St. Louis, MO). Acrylamide, bis-acrylamide, and ammonium persulfate (APS) were purchased from Thermo Scientific (Waltham, MA). Basic Green 4 (malachite green; MG) was purchased from Biosynth Carbosynth (Staad, Switzerland). N-methyl mesoporphyrin IX (NMM) was purchased from Frontier Scientific Inc. (Logan, UT). Crystal violet (CV) and cortsiol were purchased from Tokyo Chemical Industry (Tokyo, Japan). Sera-Mag™ Magnetic Streptavidin Microparticles were purchased from GE Healthcare (Chicago, IL). DNase I and S1 nuclease were purchased from Promega (Madison, WI). RNase H was purchased from Enzynomics (Daejeon, Korea). RNase A was purchased from Roche (Basel, Switzerland). Exonuclease T, T5 exonuclease, and Exonuclease V were purchased from New England Biolabs (NEB) (Ipswich, MA). Saliva (catalogue number: 991-05-F) was obtained from Lee Biosolutions (Maryland, MO). All purchased oligonucleotides and chemicals were used as received without further purification process.

### Molecular beacon in CDHP

We prepared 4 M CDHP (pH 7.4–7.8) by dissolving a CDHP powder with the proper volume 4 M NaOH solution at high temperature ∼90°C. To evaluate target mRNA responsiveness of the MnSOD-specific molecular beacon (MnSOD MB) in 2 M CDHP, we pre-mixed 10× DPBS (10 μl) with 4 M CDHP (50 μl) (pH 7.5), 1 μM MnSOD MB (2.5 μl), and 27.5 μl nuclease-free water. After vigorously vortexing of a mixed solution, the MnSOD mRNA-22 (10 μl) with varying concentrations was challenged at 37°C (final experimental condition: 1× DPBS, 2 M CDHP, 25 nM MnSOD MB, and 100 μl volume). After 5-min incubation, prepared samples were transferred to a flat-bottomed black 96-well microplate (Greiner; Kremsmünster, Austria), and the Cyanine-3 (Cy3) signal (at 575 nm) was measured by the Spark 10M microplate reader (excitation = 530 nm), with experiments conducted in triplicate. To normalize fluorescent signals, the fluorescence intensity of MnSOD MB with and without MnSOD mRNA-22 (1 μM) was set to one and zero, respectively. In the control experiment (1× DPBS), the nuclease-free water instead of 4 M CDHP was employed. When investigating the target specificity of MnSOD MB, the concentration of both single and double mismatched RNA was set to 50 nM.

### Malachite green aptamer in CDHP

To analyze the binding between MG and MG-specific aptamer in CDHP, 4X aqueous buffer (25 μl) (400 mM KCl, 20 mM MgCl_2_, and 40 mM HEPES, pH 7.4) was mixed with the 4 M CDHP (50 μl) (pH 7.5) and 1 μM MG aptamer (10 μl). Subsequently, a MG solution (15 μl) with varying concentrations was added (final experimental condition: 1× aqueous buffer, 2 M CDHP, 100 nM MG aptamer, 100 μl volume). The fluorescent signal of MG was determined with the following settings: excitation = 605 nm, emission = 643 nm. This experiment was performed in triplicate. In the control experiment without CDHP, the nuclease-free water instead of 4 M CDHP was used. To evaluate the target specificity of MG aptamer, the concentration of both MG and CV was set to 1 μM, and fluorescence spectrum from 500 to 750 nm was scanned (emission wavelength step size: 2 nm, and excitation: 450 nm) by the Spark 10M microplate reader.

### Hammerhead ribozyme in CDHP

When investigating the ribozymatic cleavage in 2 M CDHP, we first mixed 100 μM hammerhead ribozyme (0.5 μl), 100 μM Sub (0.25 μl), 500 mM Tris–HCl (pH 7.8) (1 μl), and nuclease-free water (1.75 μl), to ensure the complex formation between ribozyme and Sub. Thereafter, we prepared a CDHP solution with MgCl_2_ by mixing 5 μl 4 M CDHP (pH 7.7) and 1.5 μl 4 M MgCl_2_ for 10 min. The reaction mixture (total volume: 10 μl) of two prepared solutions was incubated at 37°C for 3 h, and then reaction sample (2 μl) was collected to be resolved on an 8M urea-20% PAGE–1× TBE gel. All gel images were acquired by using the Azure c600 (Azure Biosystems; Dublin, CA). When adjusting CDHP concentrations, we used a diluted CDHP solution instead of 4 M CDHP. In the target specificity assay, three different RNAs (Sub, N-Sub and G-Sub) were employed.

### Nucleases in CDHP

In this assay, by considering unit definition of nucleases described by the providers, excess nucleases were utilized to digest 25 pmol of nucleic acids in a 10 μl sample (2.5 μM): 1 ng RNase A (2000-fold), 5 units of RNase H (200-fold), 1 unit of DNase I (4.1-fold), 2 units of T5 exonuclease (80-fold), 10 units of exonuclease V (400-fold), and 4 units of S1 nuclease (16-fold). Because there was no unit definition of exonuclease T, we assumed that the exonuclease activity of one unit is similar to the other two exonucleases (T5 exonuclease and exonuclease V) provided by the same company (NEB). Based on this assumption, we used excess exonuclease T (5 units; 200-fold). If not mentioned separately, our experiments were conducted using above-determined amounts of seven different nucleases.

In water-only experiments, except RNase A that performed its catalytic hydrolysis in 1× DPBS, hydrolytic activities of the other six nucleases were monitored in optimal conditions recommended by providers (Enzynomics, Promega, and NEB): 1× RNase H reaction buffer (50 mM Tris–HCl, 75 mM KCl, 3 mM MgCl_2_, 10 mM DTT, and pH 8.3) for RNase H, 1× DNase I reaction buffer (50 mM sodium acetate, 280 mM NaCl, 4.5 mM ZnSO_4_ and pH 4.5) for DNase I, 1× S1 reaction buffer (40 mM Tris–HCl, 10 mM MgSO_4_, 1 mM CaCl_2_ and pH 8.0) for S1 nuclease, 1× NEBuffer™ 4 (50 mM potassium acetate, 20 mM Tris-acetate, 10 mM magnesium acetate, 1 mM DTT and pH 7.9) for three exonucleases (exonuclease T, T5 exonuclease, and exonuclease V). Because exonuclease V required ATP to facilitate the hydrolysis, 1 mM ATP was added in all exonuclease V experiments. Each optimal buffer was pre-mixed with an appropriate nucleic acid for 2 min, and then each nuclease was added at 37°C for 30 min. The negative control experiment did not include a nuclease in the sample. The reaction sample was collected, and then resolved on an 8 M urea–10% PAGE–1× TBE gel.

To evaluate nuclease activities in CDHP, a nucleic acid was pre-mixed with the 4 M CDHP (pH 7.5) for 1 min, and a suitable 10× reaction buffer (10× DPBS, 10× RNase H reaction buffer, 10× DNase I reaction buffer, 10× S1 reaction buffer, and 10× NEBuffer™ 4) and a nuclease-free water were added to this mixture. After vigorously pipetting prepared solutions, a nuclease was finally challenged and then incubated at 37°C for over 24 h (Final reaction condition: 2.5 μM nucleic acid, 2 M CDHP, 1× optimal buffer, the appropriate amount of each nuclease, and 10 μl volume). During the incubation, a reaction sample was collected at proper time intervals (0.5, 6, 12 and 24 h), and then resolved on the 8 M urea–10% PAGE-1× TBE gel.

### Functions of nucleic acids mixed with nuclease-containing samples in CDHP

In 100 μl nuclease cocktail, we mixed seven different nucleases: 10 ng RNase A, 5 units RNase H, 10 units exonuclease T, 1 unit DNase I, 20 units T5 exonuclease, 20 units exonuclease V and 40 units of S1 nuclease. This nuclease cocktail was always freshly prepared prior to use. When performing nuclease cocktail-included experiments with three different FNAs, one tenth of the experimental volume was occupied by the nuclease cocktail. For instance, when we analyzed MG-specific aptamer with 2 M CDHP (final volume: 100 μl) in the nuclease cocktail, 10 μl nuclease cocktail was added instead of nuclease-free water (experimental condition: 1× aqueous buffer, 2 M CDHP, 100 nM MG aptamer, and 10 μl nuclease cocktail). Thereafter, the mixture was immediately incubated at 37°C for 3000 min, and then we analyzed fluorescent signals. When we conducted the control experiment with no CDHP, the prepared sample was incubated for 30 min at 37°C. All these experiments were performed in triplicate.

To evaluate performance of the MnSOD MB in saliva, we first prepared a 60 μl sample (1.67× DPBS, 3.33 M CDHP, 41.66 nM MnSOD MB) with MnSOD mRNA-22 at varying concentrations, and the volume of this mixture was reduced down to ∼50 μl by using Micro-Cenvac (N-Biotek; Bucheon, Korea). To this sample (∼50 μl), the same volume of 100% non-treated human saliva was added to set the concentration of CDHP and saliva as 2 M and 50%, respectively. After preparing the reaction mixture, it was immediately incubated at 37°C for 3000 min (in the case of sample with no CDHP, we performed 30-min incubation). After incubation, we measured the Cy3 signal of each sample as described above. When we analyzing the MG-specific aptamer in saliva, we prepared 65 μl experimental sample (1.54× selection buffer, 3.08 M CDHP, 154 nM MG aptamer) with varying concentrations of MG, and then reduced the reaction volume down to ∼50 μl. The same volume of 100% saliva was added, and the final mixture was incubated at 37°C for 3000 min. Finally, the fluorescent signal of MG was determined (at 643 nm). In the case of the hammerhead ribozyme assay in saliva, we prepared a reaction mixture at 37°C with double concentrations of all reagents under the reaction condition described above, and then the same volume of 100% non-treated human saliva was added (final concentration of CDHP and saliva: 2 M and 50%, respectively). The mixed solution was incubated at 37°C for 3000 min.

## Results

**Figure 1.**
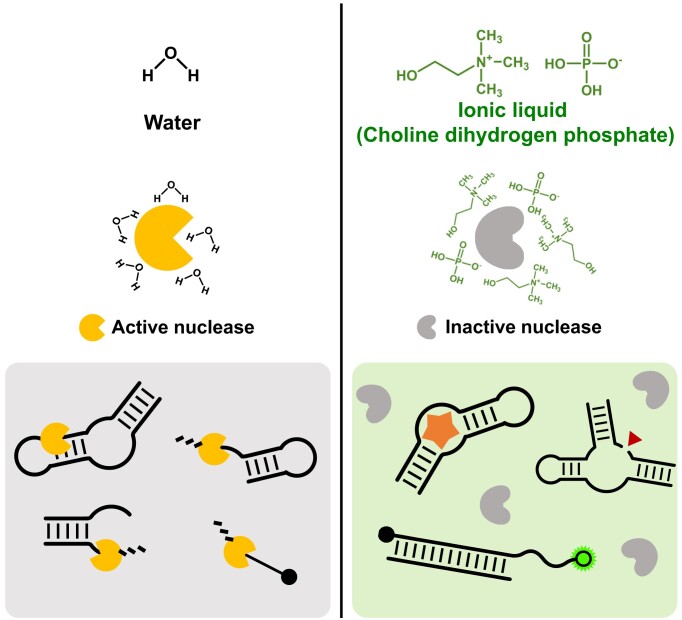
Different behaviors of nucleases and FNAs in two different cages (water and CDHP ionic liquid). In water, nucleases are catalytically active to rapidly digest nucleic acids, with no chance of active folding of FNAs (left). However, the CDHP cage forces all kinds of nucleases to be fully inactivated in a non-hydrolytic environment; as the FNAs still maintain their functional structures in the CDHP, they can be hydrolytically resistant and functionally active at the same time (right).

Unlike water, ionic liquids guide FNAs and nucleases to behave differently (Figure 1). As the water allows the nucleases to be enzymatically active, the phosphodiester bonds within the FNAs are rapidly cleaved by enzyme-driven hydrolysis, leading to the permanent loss of their desired functions (Figure 1, left). However, in the CDHP ionic liquid, both spontaneous and biological degradation of nucleic acids can be readily blocked or retarded while the FNAs sustain their native conformations; i.e. aptamers and nucleozymes fold into unique binding- and catalytic-competent states to recognize cognate targets or to provoke biochemical reactions (Figure 1, right). Accordingly, without complicated chemical modifications or temperature controls, native forms of the FNAs in the CDHP hold unexplored potential to be highly active even in nuclease-containing clinical specimens or undesirable contaminations.

### Conserved functions of CDHP-caged nucleic acids

**Figure 2.**
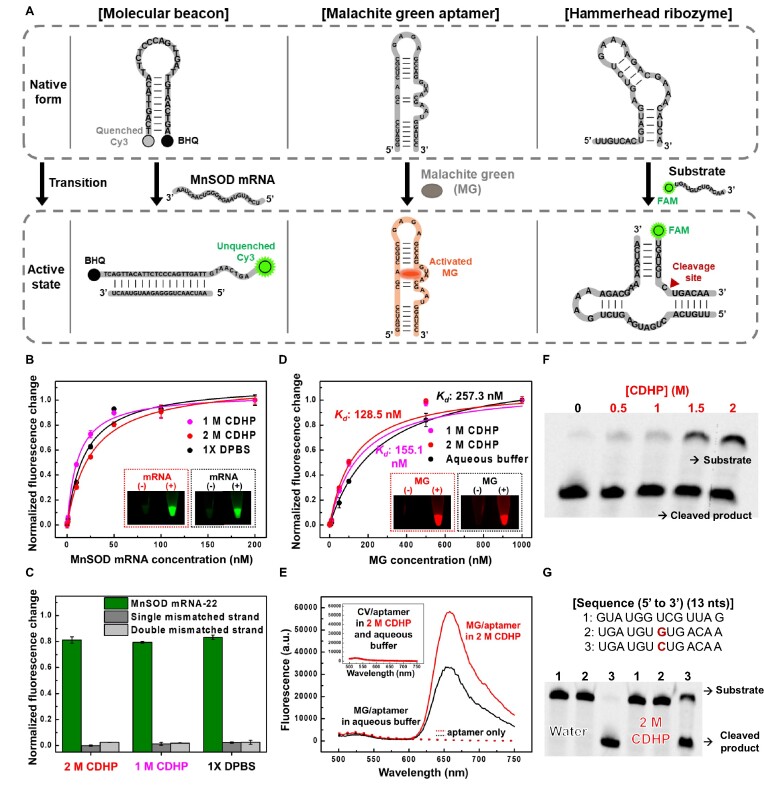
Confirmation of active FNAs in the CDHP cage. (**A**) Three different FNAs (MnSOD mRNA-sensing MB, MG aptamer and hammerhead ribozyme) and their pre-defined molecular functions. In response to cognate targets or substrates, the FNAs can be activated to perform specific target recognition or substrate cleavage, synchronized with detectable fluorescent changes. (**B**) The MnSOD MB-based mRNA detection in CDHP (1 and 2 M) and 1× DPBS. Like 1× DPBS, the CDHP allowed RNA:DNA hybridization to induce a large conformational change of MnSOD MB in emitting a strong fluorescent signal. As a result, we observed similar signaling patterns in a target concentration-dependent manner. (**C**) Conserved target specificity of MnSOD MB. Either in CDHP or in 1× DPBS, the MnSOD MB could discriminate its target RNA from single or double mismatched non-target RNAs. (**D**) Aptameric MG recognition and fluorescent increases in CDHP (1 and 2 M) and aqueous buffer. In the CDHP, the MG-specific aptamer could recognize its target fluorogen to perform binding-induced fluorescent enhancing, even exhibiting lower *K*_d_*s* than in the aqueous buffer. (**E**) Conserved target specificity of MG-specific aptamer. Unlike MG, its structural analog, CV, could not be bound to the MG-specific aptamer without fluorogen stabilization, leading to no increase in fluorescence either in CDHP or in aqueous buffer. (**F**) Ribozyme-mediated substrate cleavage at different CDHP concentrations. The hammerhead ribozyme could perform the desired phosphoryl transfer reaction in the CDHP with slightly lower yields than in water. (**G**) Conserved substrate specificity of hammerhead ribozyme. The ribozyme selectively cleaved its target substrate both in water and 2 M CDHP.

To thoroughly scrutinize whether FNAs can perform their desired functions in the CDHP cage, we chose three different types of FNAs (Supplementary Table S1): manganese superoxide dismutase (MnSOD) mRNA-sensing molecular beacon, malachite green (MG) aptamer, and hammerhead ribozyme (Figure 2A). First, we designed the MnSOD mRNA-sensing molecular beacon that induces close proximity between 3′-labeled Cyanine-3 (Cy3) and 5′-labeled Black Hole Quencher 2 (BHQ2) (Figure 2A, left). The hairpin-shaped DNA can specifically hybridize with the MnSOD mRNA relevant to tumor metastasis (40) and apoptotic signaling (41). This hybridization causes the separation of Cy3 and BHQ2, thereby generating an unquenched fluorescent signal (at 570 nm) in a target concentration-dependent manner. Second, the MG aptamer (42), one of the fluorescent light-up RNA aptamers, can recognize the MG molecule (Figure 2A, middle), a triphenylmethane fluorophore that displays an extremely low quantum yield with no complex formation; upon aptamer binding, the fluorescence of MG can be activated with the increase of fluorescent signal (at 648 nm) up to 2000-fold (43). Third, the hammerhead ribozyme, in complex with a 5′-FAM-labeled substrate by RNA:RNA hybridization, enables the site-specific cleavage of phosphodiester bond (Figure 2A, right). When the ribozyme performs its catalytic reaction, the substrate is split into two RNA fragments, which can be readily confirmed by gel electrophoresis.

Our mRNA-sensing molecular beacon (MnSOD MB) was capable of detecting its specific target mRNA in the CDHP cage, relying on an RNA:DNA hybridization-driven conformational change (Figure 2B, C). When a 22-nt-long fragment of human MnSOD mRNA (MnSOD mRNA-22) was challenged with our designed MB in 2 M CDHP (Figure 2B, red dotted box), the evident target-induced increase in Cy3 fluorescent signal was observed similar to that in 1× Dulbecco's phosphate buffered saline (DPBS) (black dotted box). Even with 1 and 2 M CDHP (magenta and red, respectively), the MnSOD MB was fully responsive to report MnSOD mRNA concentrations like that in the isotonic DPBS (black). For investigation of ion effects, we measured the limit of quantification (LOQ) of MB in five different conditions (Supplementary Figure S1): CDHP (1 and 2 M), 1× DPBS, and NaCl solutions (1 and 2 M) that can give the high ionic strength like the CDHP environment. As a result, we identified that the LOQ value of MB was highly consistent in five different buffers; along with the types of cation (choline and Na^+^) and anion (phosphate and Cl^–^), their concentrations (1 and 2 M) did not alter the target sensitivity of MB. In addition to the specific base-base interactions of DNA duplexes (30,44), those of RNA:DNA duplexes in the CDHP were also confirmed as the MB discriminated the MnSOD mRNA-22 from single mismatched and double mismatched strands (Figure 2C).

In the CDHP cage, nucleic acids enable aptameric target recognition, sometimes with stronger affinities than in water (Figure 2D, E). Even though aptamers rely on various interaction forces, including electrostatic attraction, hydrogen bond, and hydrophobic association in target binding (45), we observed the successful complex formation between the MG aptamer and the MG in 2 M CDHP (Figure 2D, red dotted box), exhibiting a MG-dependent fluorescence increase like that in the aqueous buffer (100 mM KCl, 5 mM MgCl_2_, 10 mM Na-HEPES, pH 7.4) (46) (black dotted box). When we measured equilibrium dissociation constants (*K*_d_s) of the MG aptamer with or without CDHP (Figure 2D), the *K*_d_ in 2 M CDHP (128.5 nM) was even lower than that in the aqueous buffer (257.3 nM); the improved affinity to the target MG would be attributed to the low water activity of ionic liquid (47), facilitating the dehydration process of the MG in complex with its aptamer (48). Importantly, the MG aptamer was proven to be target-specific even in CDHP (Supplementary Figure 2E and S2) from the two key observations that the MG aptamer did not stabilize the crystal violet (CV), a structurally similar triphenylmethane dye to the MG (Figure 2E, inset), and a scrambled RNA failed to increase the MG fluorescence with no binding event (Supplementary Figure S2).

The aptameric target recognition in CDHP is not limited for either RNA aptamers or for small molecule targets; even DNA aptamers can exhibit their binding affinities to different kinds of cognate targets. For example, the G-quadruplex-structured PS2.M aptamer (49) successfully recognized two different small molecules, N-methyl mesoporphyrin IX and thioflavin T, without much differences of *K*_d_ values in optimal buffers and in 2 M CDHP (Supplementary Figure S3A). Moreover, the tight complex formation between a DNA aptamer and a large protein was also achievable in 2 M CDHP, evidenced by successful streptavidin recognition of St-21–1 aptamer (Supplementary Figure S3B) (50).

Active folding of nucleic acids in the CDHP cage permits catalyzing biochemical reactions, such as a phosphoryl transfer reaction (Figure 2F, G). Theoretically, when a hammerhead ribozyme is self-assembled with its substrate to form three stems by Watson-Crick pairings (Supplementary Figure S4A), there can be site-specific substrate cleavage by RNA-catalyzed transfer of phosphoryl group. As we confirmed the increased thermal stability of A:U pairs in RNA duplexes (Supplementary Figure S5), similar to that of A:T pairs in DNA duplexes with the CDHP (30,44), we newly designed a CDHP-compatible hammerhead ribozyme by insertion of intended A:U pairs (Supplementary Figure S4B). The engineered ribozyme readily cleaved its substrate in the CDHP cage (Figure 2F); while the catalytic yield was slightly lower than in water, the reaction itself did not strongly depend on the CDHP concentrations (Supplementary Figure S6). The ribozymatic cleavage always demanded both the hammerhead ribozyme and Mg^2+^, confirming their CDHP-independent specific interaction (Supplementary Figure S7). Moreover, the ribozyme remained substrate-specific in cleavage (Figure 2G), forcing undesired substrates (e.g. an unhybridized RNA (lane 1) and a C-to-G replaced one at the cleavage site (lane 2) (51) to be uncleaved, regardless of the presence of CDHP.

### Nuclease-resistant CDHP-caged functional nucleic acids

**Figure 3.**
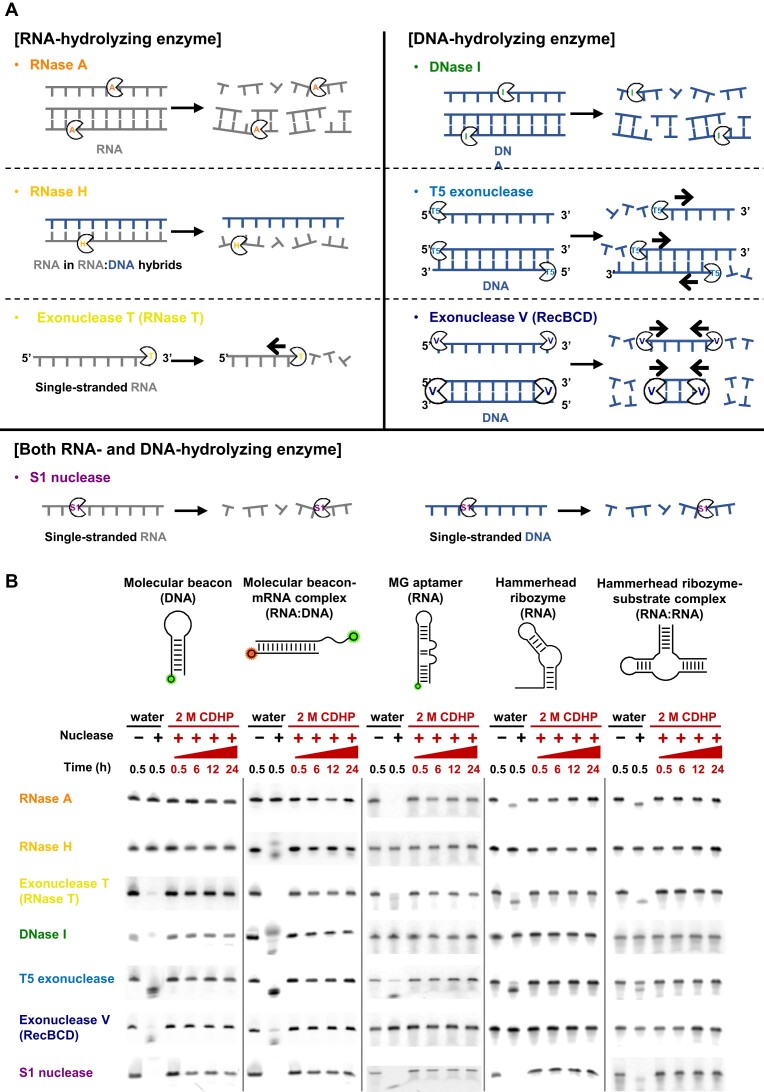
Hydrolysis-resistant FNAs in the CDHP cage against various nucleases. (**A**) Seven different nucleases (RNase A, RNase H, exonuclease T, DNase I, T5 exonuclease, exonuclease V and S1 nuclease) and their distinct nucleic acid specificities. (**B**) CDHP-mediated protection of FNAs against the different nucleases. In water, all the nucleases fully digested their target nucleic acids within 30 min (lanes 1–2), but in 2 M CDHP, all the chosen FNAs were well conserved with different molecular structures over a day at 37°C (lanes 3–6) as all kinds of nucleases were inhibited effectively. Oligonucleotides in this assay include some additional modifications (Supplementary Figure S8) for visualization of their nucleolytic degradation. In monitoring the hydrolysis of molecular beacon-mRNA complex, the fluorescence of MnSOD MB-Cy3 was visualized in gel electrophoresis, except for RNase H; for the RNase H-based digestion, that of MnSOD mRNA-22-Cy5 was visualized instead, in hybridization with MnSOD MB-Cy3.

In protecting conformational and functional integrities of FNAs, we next investigated whether CDHP can serve as a universal inhibitor against a variety of ubiquitous nucleases (Figure 3A). Assuming the threat of enzyme-mediated nucleic acid degradation, we prepared seven different nucleases with distinct specificities (52–55) (Figure 3A): RNase A, RNase H, exonuclease T (RNase T), DNase I, T5 exonuclease, exonuclease V (RecBCD), and S1 nuclease. As adventitious contaminants in laboratories, the naturally occurring nucleases pose a serious risk of hydrolytic damage to many kinds of FNAs; depending on the active folding structures of several FNAs, a number of different positions, such as single-stranded overhangs and double helical domains, were identified to be vulnerable to the different nucleases (Supplementary Table S2).

Due to effective inhibition of nuclease activities in CDHP, full-length FNAs were well conserved for a day at 37°C, even with seven different nucleases (Supplementary Figures 3B and S8). With no CDHP, nucleases were catalytically active, digesting all the FNAs within 30 min completely (Figure 3B, lanes 1–2). However, regardless of different folding, all the chosen forms of FNAs were remarkably resistant to nuclease-mediated hydrolysis over a day in 2 M CDHP (lanes 3–6); for instance, the hairpin-shaped DNA MB with a blunt end and the mRNA-bound one with a 3′ overhang both remained intact in the presence of various DNases. Importantly, the CDHP-mediated protection of nucleic acids is clearly attributed not to protein denaturation, but to hydrolysis inhibition, which was evidenced by the activity recovery of nucleases when we diluted nuclease-containing 2 M CDHP solutions with excess water (Supplementary Figure S9). This result supports our claim that the CDHP-mediated nuclease inhibition is apparently the cage effect by kicking out water molecules, the main reactants in hydrolysis, around nucleic acids.

Importantly, we demonstrated for the first time that nucleases exhibit CDHP concentration-dependent nucleolytic activities, and the threshold concentrations of CDHP in inhibition can be varied for the types of nucleases (Supplementary Figures S10 and S11). Theoretically, when a catalytically active site of nuclease is assembled with a target nucleic acid, the access of water molecules is essential for the desired hydrolytic activity of the nuclease. As the water and CDHP compete each other to be around the nucleic acid, the degree of CDHP-mediated water molecule displacement can be varied with CDHP concentrations. Moreover, nucleases of different assembling structures and hydrolytic mechanisms display different affinities to their own targets and water molecules, inferring that the desired CDHP concentrations would be varied to inhibit different nucleases. We indeed identified that nuclease activities depend on CDHP concentrations; some nucleases (S1 nuclease and exonuclease T5) were highly susceptible to the presence of CDHP with no hydrolysis activities even at <400 mM CDHP. We also found that the minimum CDHP concentration to effectively inhibit all the seven nucleases is ∼1.5 M.

Unlike conventional strategies, our CDHP-mediated nucleic acid protection does not depend on types of nucleases. Currently, it cannot be standardized to retard all the kinds of nuclease-mediated degradation because several notorious nucleases are highly active even after the well-known inhibition treatments (*e.g*. heat inactivation (56), divalent cation chelation (57), chemical denaturant (58) and organic solvent addition (59,60), and increased ionic strength (61)). For example, reversibly foldable RNase A and T5 exonuclease are hardly to be inactivated with high temperature (62,63), and a few classes of DNase and RNase are not intimidated by chelating agents, relevant to their metal ion-independent cleavage mechanisms (54). However, their nucleolytic activities can be readily suppressed with non-hydrolytic microenvironments, suggesting that our CDHP-based simple strategy holds great potential to universally inhibit nucleases even in unknown biological or contaminated samples.

**Figure 4.**
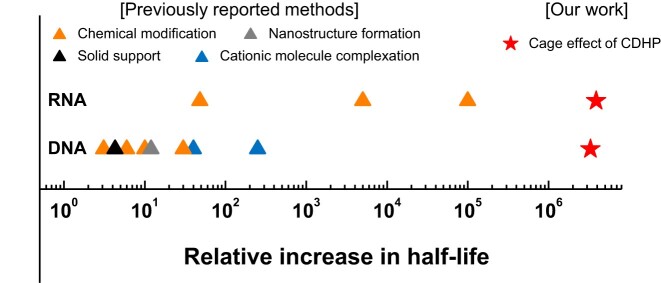
Relative increase in DNA and RNA half-lives among a variety of nucleic acid protection strategies. The relative increase in half-life is calculated by dividing the half-life of protected nucleic acids by that of unprotected ones in the presence of nucleases (Supplementary Table S3). Compared to previously reported strategies (blue), our CDHP cage is exceptionally effective in extending half-lives of nucleic acids (red). The largest relative increases of RNA and DNA half-lives are against RNase A and DNase I, respectively (Table 1).

The exceptional protective effect of CDHP should be further emphasized; irrespective of nuclease types, the FNAs remained intact even after 1-month incubation at 37°C. To scrutinize half-lives of our oligonucleotides either in water or 2 M CDHP, we monitored nucleolytic degradation in a time-dependent manner (Supplementary Figures S12 and S13). In water, active nucleases caused their target nucleic acids to disappear shortly (Table 1 and Supplementary Figure S12). For example, against RNase A, Exonuclease T, and S1 nuclease with RNase activities, half-lives of MG-specific RNA aptamers were less than 10 sec, and the longest half-life was just 144.4 s for DNA MBs in the presence of DNA-hydrolyzing exonuclease V. In 2 M CDHP, however, the half-lives of all the chosen FNAs were remarkably extended against all the chosen nucleases with negligible degradation for 1 month (Table 1 and Supplementary Figure S13). When assuming the first order decay kinetics (64), the half-lives of DNAs and RNAs were calculated to be longer than 5 months even at 37°C. Against DNase I, previous DNA complexation with cross-linked oligolysines reported a 250-fold increased half-life from 16 min to 66 h (65) (Supplementary Table S3), but the use of 2 M CDHP demonstrated the greater improvement of relative increase in half-life, by ∼3 300 000 times. Although the multiplexed incorporation of chemical modifications (2′-NH_2_ or 2′-C-allyl modifications with 3′-3′-linked nucleotide caps) allowed the modified RNAs to yield ∼100000-fold half-life increase in a sample with RNases (11), the simple CDHP cage with no heavy RNA modification was even ∼38 times superior to increase the RNA half-life, with the improvement of relative increase in half-life against RNase A by ∼3 800 000 times. By recording the longest DNA and RNA half-lives, to the best of our knowledge, our CDHP-mediated nucleic acid shielding would be the most powerful to resist many different nuclease-driven degradation (Figure 4 and Supplementary Table S3).

**Table 1.** The half-lives of nucleic acids against various nucleases either in aqueous buffer or in 2 M CDHP

Nuclease	Half-life in water	Half-life in 2 M CDHP^d^	Relative increase in half-life
RNase A^a^	<5 s	224.3 days	>3 875 904
RNase H^b^	37.9 s	267.6 days	610 043
Exonuclease T^a^ (RNase T)	8.4 s	636.1 days	6 542 742
DNase I^c^	6.0 s	229.3 days	3 301 920
T5^c^ exonuclease	56.0 s	730.2 days	1 126 594
Exonuclease V^c^	144.4 s	304.6 days	182 253
S1 nuclease^a^	7.5 s	158.7 days	1 828 224

^a^MG aptamer-FAM was employed. ^b^MnSOD mRNA-22-Cy5, hybridized with MnSOD MB, was employed. ^c^MnSOD MB-Cy3 was employed. ^d^Assuming the first order decay kinetics, half-lives of RNAs and DNAs are calculated based on Supplementary Figure S13. The relative increase in half-life is the half-life of nucleic acids in 2 M CDHP divided by that in water.

### CDHP-mediated active folding of FNAs with full resistance to nucleases

**Figure 5.**
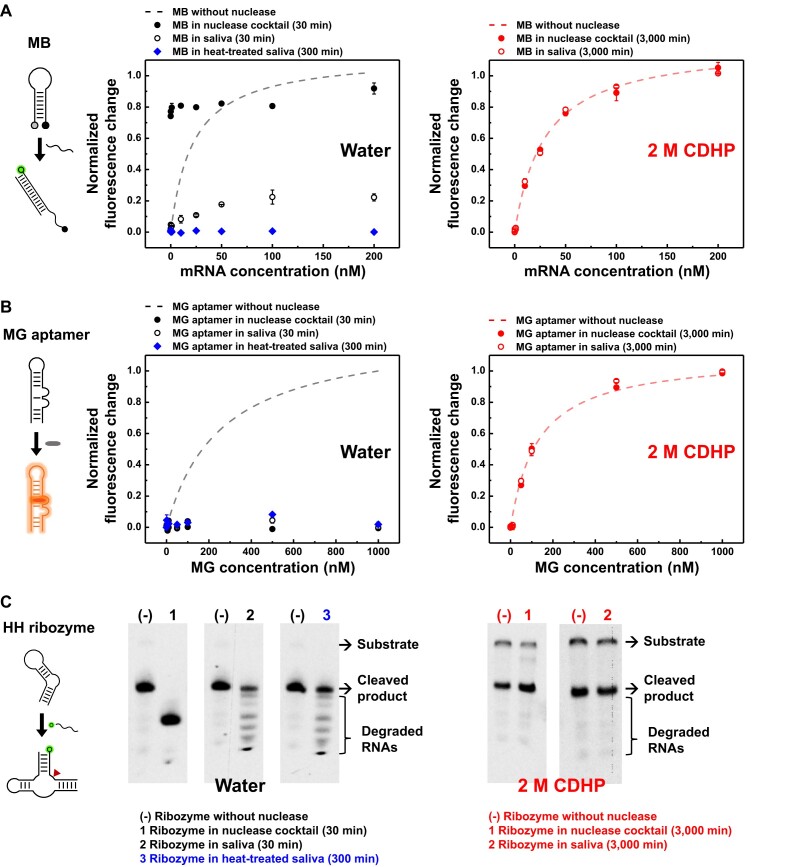
CDHP-mediated shielding of active FNAs in nuclease cocktails and human saliva. (**A**) MB-based mRNA detection with or without 2 M CDHP. Both nuclease cocktails and saliva (non- or heat-treated) permitted nucleolytic digestion of MnSOD MB or MnSOD mRNA-22 without CDHP, relevant to false-positive or true-negative detection errors, respectively (left). With 2 M CDHP, however, both MnSOD MB and MnSOD mRNA-22 were fully shielded even in the presence of undesired and unknown nucleases during 3000 min incubation at 37°C, observing desired fluorescent signals in a target concentration-dependent manner like the intact MB with no nuclease (right). (**B**) Target recognition of MG-specific aptamers with or without 2 M CDHP. Without CDHP, high RNase activities in nuclease cocktails and saliva (non- or heat-treated) caused full hydrolysis of MG-specific RNA aptamers, and no aptamer-bound stabilization of MGs was detected with observation of true-negative fluorescent signals (left). However, use of 2 M CDHP effectively inhibited the RNase activities over 3000 min at 37°C, allowing the MG-specific aptamer to perform binding-induced fluorescent enhancing in a MG concentration-dependent manner with no decrease in affinity (right). (**C**) Ribozyme-mediated substrate cleavage with or without 2 M CDHP. The absence of CDHP revealed the undesirable loss of nucleic acids, including cleaved products, both in nuclease cocktails and human saliva (non- and heat-treated) (left). On the other hand, the CDHP cage allowed desired substrate-specific cleavage reactions irrespective of the presence of various nucleases, and both uncleaved substrates and cleaved products were well conserved even after 3000 min incubation at 37°C (right). For all the experiments, the heat treatment of saliva was performed at 90°C for 15 min.

It is not trivial that nucleic acids assays can be unsuccessful, because of undesired and unknown nucleases in biological fluids or contamination-suspicious samples. To simulate possible nuclease contaminations, we prepared a nuclease cocktail by simply mixing the seven nucleases, in addition to human saliva, an attractive diagnose medium for clinical studies (66). In a sample with nucleases, the three different FNAs failed to display desired molecular functions due to their undesired degradation (Figure 5A–C). When the MnSOD MB and its target RNA were incubated together in the nuclease cocktail, both strands were fully digested within 30 min, eventually releasing the Cy3 fluorophore that induced a false-positive detection error (Figure 5A, solid black circle). Moreover, due to thermostable and high RNase activities (Supplementary Figure S14) (67), both 50% non- and heat-treated saliva elicited predominance of RNA digestion, thereby causing a true-negative error in MB-based mRNA detection (Figure 5A, empty black circle and solid blue diamond). Similarly, both the aptameric target recognition and the ribozymatic product formation suffered from the multifaceted hydrolysis problems in the samples with various nucleases (Figure 5B, C, left).

As liberated from the risk of digestion and the loss of function, all the FNAs in the CDHP cage performed the desired target recognition and catalytic reaction both in the nuclease cocktail and in the human saliva. With 2 M CDHP, all the nuclease activities were successfully inactivated, thereby permitting faultless and quantitative MB-based mRNA detection (Figure 5A, right); even after 3000 min incubation at 37°C either in the nuclease cocktail or in the human saliva, the MB emitted a desired fluorescent signal in a target concentration-dependent manner without signal loss and decrease in sensitivity. Moreover, even a minor strand damage is fatal for proper folding of aptamers, but the use of 2 M CDHP was confirmed to fully secure the MG-specific aptamers against the multifaceted nuclease activities, preserving the full MG fluorescence (Figure 5B, right).

Importantly, the heat inactivation of saliva, the most widely used method to denature proteins, was unsuccessful to prevent nuclease-driven RNA hydrolysis (Figure 5A–C, blue). Despite the abundance of thermo-stable RNases in the human saliva, however, the CDHP cage facilitated the desired ribozymatic reactions with no nucleolytic degradation (Figure 5C, right). Regardless of the presence of the nuclease cocktail and the human saliva, the hammerhead ribozymes successfully underwent the RNA substrate-specific cleavage; even after 3000 min incubation at 37°C, both the uncleaved substrate and the cleaved product were readily observed with their exact length, which was attributed not by indiscriminate nucleolytic degradation, but by site-specific ribozymatic cleavage (Figure 5C, right).

CDHP-caged FNAs can be practically useful even in real biological samples due to their active molecular functions with no hydrolytic damage (Supplementary Figure S15). Recently, human saliva has proven to include diverse biomarkers for many different diseases (68), such as cancer and viral infection, and among the salivary biomarkers, cortisol is clinically important for diagnosis of diseases (*e.g*. neurodegeneration and Cushing syndrome) and even stress levels (69,70). In a previous study, an aptamer-integrated biosensor has been developed to detect the cortisol in saliva (71), but it inevitably required the removal of salivary proteins, including nucleases, to avoid the significantly impaired sensitivity in detection. However, our rationally designed aptasensor successfully reported the presence of cortisol using fluorescent signals in non-treated 100% human saliva (Supplementary Figure S15A). In the clinically accepted range of the cortisol concentrations from 1 to 10 ng/ml, cortisol-specific and concentration-dependent fluorescent signaling was observed both in an aqueous buffer and in 2M CDHP (Supplementary Figure S15B). Importantly, the limit of detection (LOD) was calculated to 1.20 ng/ml in the 2 M CDHP, which is even lower than that in the aqueous buffer (1.45 ng/ml). Indeed, the CDHP cage effect was practically useful for the direct usage of the cortisol-specific aptasensor; the presence of 2 M CDHP successfully enabled the quantitative detection of salivary cortisol even after 3000 min incubation at 37°C, attributed to the fully conserved affinity of the aptasensor against the presence of nucleases (Supplementary Figure S15C). In contrast, when the aptasensor was exposed to the cortisol-containing human saliva without CDHP, it malfunctioned immediately, making the reported cortisol concentrations neither accurate nor reproducible. Once again, the practical usage of FNAs can be readily achieved with the support of CDHP cage in clinically and biologically important fluids with no pretreatments, which would be otherwise significantly challenging with the presence of active nucleases.

## Discussion

Despite the presence of undesired and unknown nucleases, FNAs have demonstrated to perform pre-defined molecular functions since the cage effect of CDHP exclusively allows their active folding with no nucleolytic degradation; against different types of nucleases, their half-lives could be extended to ∼2 years even at 37°C in the hydrolytically unfavorable situation. It was also interesting that small molecule-binding aptamers, such as MG-specific RNA aptamer and PS2.M DNA G-quadruplex, could retain their inherent affinities and specificities to cognate fluorogen ligands in the CDHP, leading to binding-induced fluorescence enhancing like in the water. Importantly, the aptamers could be readily engineered to mimic the MBs that perform binding-induced structure-switching. For example, the ATP aptamer was chemically modified to induce target-specific close proximity between 5′ FAM and 3′ BHQ1 (72), and as a result, the presence of ATP, the nonfluorescent ligand, was specifically reported even in the CDHP in a concentration-dependent manner (Supplementary Figure S16). All the observations reveal that with no risk of hydrolysis, a variety of FNAs could be systematically combined to create more complex, yet highly useful molecular functions, such as ligand-induced chemical reactions (2), chemically regulated molecular recognition (73), and stimuli-responsive structural assembly and disassembly (74).

To induce dehydration-driven hydrolysis inhibition, the formation of CDHP cage would cause nucleic acids to be isolated from water molecules, which are otherwise localized at the vicinity of phosphate groups and minor grooves (75). First, due to the high ionic strength, the CDHP with excessive choline cations can fully cover the negatively charged phosphate groups of nucleic acids during neutralization, unless the backbone would be surround by hydrated or aqueous cations; indeed, the negative charge of both DNAs and RNAs was fully screened in high-concentration CDHP as the choline cations are in complex with the phosphate groups (Supplementary Figure S17A). Second, the CDHP can intrude into the minor groove of nucleic acids to liberate the previously bound water molecules. Some fluorescent dyes (SYBR Green I and methylene blue) can be intercalated into the minor groove of nucleic acids by liberating pre-bound water molecules, but their minor groove binding was even further mitigated in 2 M CDHP due to choline substitution (Supplementary Figure S17B) (30,34), which is also relevant to the decreased affinity of ATP aptamers that construct their adenosine binding pockets by exploiting the minor groove edges of aptamer (76) (Supplementary Figure S16). From these two observations, it is concluded that in high-concentration CDHP, the water molecules could be discharged from their original binding sites, resulting in localized dehydration for hydrolysis resistance of nucleic acids.

Many kinds of CDHP-caged aptamers, however, exhibited improved affinities to their own targets (Supplementary Figures 2B and S3) with three plausible reasons as described below.

First, an excessively large amount of choline cations in the CDHP could be beneficial to enhance the aptameric target recognition. Polyanionic nucleic acids usually demand the presence of cations to overcome their charge-charge repulsions during their stable secondary and tertiary structure formation (77), and the presence of the excess cations occasionally supports the negatively charged aptamers to show stronger affinities to cognate targets (78). For example, in our experiments (Supplementary Figure S3B), the streptavidin-binding aptamer exhibited the lowered *K_d_* value (75.4 nM) to its protein target in 2 M NaCl than that in a low-salt buffer (94.5 nM in 1× PBS). Likewise, 2 M CDHP lowered the *K*_d_ value (70.0 nM) with the excess of choline cations.

Second, it is well known that inclusion of ionic liquids, including CDHP, lowers the water activity in an aqueous solution due to high ionic strengths and low water contents (47), which can be highly advantageous for dehydration-driven aptameric target recognition. For instance, we demonstrated that as the target binding site of MG aptamer should be dehydrated before the capture of MG (45), it was observed that the *K*_d_ value in CDHP (128.5 nM) became lower than that in the aqueous buffer (257.3 nM, Figure 2D).

Third, a crowding condition of CDHP may elicit an excluded volume effect that allows the aptamer folding equilibrium to be a structurally compacted state with improved target binding (79,80). Diverse cosolutes (e.g. proteins, nucleic acids, synthetic polymers, and peptides) have markedly accelerated molecular compaction as crowding agents (81–84), forcing some aptamers (e.g. MG aptamer (85) and human telomeric G-quadruplex (86)) to possess pre-folded compact structures which cannot normally be attained under target-deficient conditions. When two different FNAs, the MG aptamer and the human telomeric G-quadruplex, were labeled with Cy3 and Cy5 as fluorescence resonance energy transfer (FRET) pairs, we monitored their CDHP concentration-dependent structural transitions by analyzing FRET efficiencies (Supplementary Figure S18). As a result, it was found that in the absence of desired targets (MG and potassium ion, respectively), both the FNAs displayed more compacted structures in CDHP, implying that the CDHP cage may facilitate aptamer-target complexation by encouraging the aptamers to readily construct their target-bound folding conformations.

In nucleic acid protection, it should be emphasized that the CDHP-mediated simple strategy is superior to previously reported ones with two major reasons as described below.

First, the CDHP cage-based protection of nucleic acids does not depend on the types of nucleases. The well-known inhibition treatments (*e.g*. heat inactivation, catalytic ion chelation, and chemical denaturation) cannot be standardized to retard all kinds of nuclease-mediated degradation because several notorious nucleases are still resistant to the treatments. We experimentally confirmed that commercially available RNase inhibitors, which are widely utilized to protect RNAs, failed to block RNase A-driven RNA degradation even with the excessive amount of the inhibitors (Supplementary Figure S19). However, our CDHP-mediated nucleic acid protection is nuclease type-independent, exhibiting full hydrolysis resistance against diverse nucleases (Figure 3) and even in biological solutions (Figure 4 and Supplementary Figure S15).

Second, the CDHP-mediated hydrolysis inhibition is applicable for all the types of nucleic acids without chemical or physical modifications. Some chemical modifications, such 2′-*O*-methylation (18), nucleobase modification (87), and 3′-3′ terminus linkage (20), are known to offer the hydrolysis resistance to nucleic acids, but they are heavily dependent from nucleic acid types, sequence compositions, and even modification positions. For example, 2′-hydroxyl group modifications are not applicable for DNAs, and different methylation positions of five natural nucleobases (G, C, A, and T/U) cause different levels of nuclease susceptibility for the modified nucleic acids (88). Moreover, internal chemical modifications, which are typically significantly expensive, require special phosphoramidite monomers for automated oligonucleotide synthesis, but there are not many types of available modifications to block nuclease-mediated degradation. Besides, even if the modifications seem to be successful, they frequently alter the pre-defined conformations and functions of original nucleic acids (89,90). In CDHP, however, the nucleic acids are intact, irrespective of their structures, lengths, functions, and even types, making the simple and widely applicable CDHP cage more advantageous than many other methods in nucleic acid protection.

Compared to many other non-canonical solvents, furthermore, the CDHP is an unrivaled one for nucleic acid-based assays. Previously, several organic liquids (*e.g*. dimethyl sulfoxide, dimethylformamide, acetonitrile, and various alcohols) have been used to reduce hydrolytic activities of nucleases, but it was frequently observed that the levels of RNase and DNase activities were slightly diminished or unaffected at all (58–60,91,92). Importantly, nucleic acids in the organic solvents are destabilized without their native folding structures, accompanying undesirable non-specific interactions (93,94), which is attributed to the solvation of hydrophobic nucleobases unlike in water. However, the CDHP, one of ionic liquids, were demonstrated for the first time to serve as a universal nuclease inhibitor for all kinds of nucleolytic activities and even the spontaneous hydrolytic ones when the active folding structures of nucleic acids, such as aptamers and nucleozymes, maintained their complex molecular-level interactions with their specific target ligands and substrates. Moreover, the CDHP has been proven to exhibit excellent biocompatibility as this ionic liquid is composed of a choline cation and a phosphate anion, both of which are biologically abundant (39,95). As encouraged by the recent report that human cells are highly viable in 2.5 M CDHP, we envision to practically prolong the activities of versatile nucleic acids for both *in vitro* and *ex vivo* applications, which is still inaccessible due to the lack of widely applicable strategies that enable universal inactivation of nucleases, but permit useful functions of nucleic acids at the same time.

## Supplementary Material

gkad1093_Supplemental_File

## Data Availability

The data underlying this article are available in the article and in its online supplementary material. Further data underlying this article will be shared on reasonable request to the corresponding author.
