# Supplementary material for: Ionic liquid-caged nucleic acids enable active folding-based molecular recognition with hydrolysis resistance
Source: Nucleic Acids Res. 2023 Nov 22;52(1):73–86. doi: 10.1093/nar/gkad1093 (PMC10783497; doi:10.1093/nar/gkad1093)
Supplement: gkad1093_Supplemental_File [file gkad1093_supplemental_file.pdf]

Supporting Information for

Ionic liquid-caged nucleic acids enable active folding-based molecular recognition with hydrolysis resistance

Byunghwa Kang[1,†], Soyeon V. Park[1,†], and Seung Soo Oh[1,2,*]

[1] Department of Materials Science and Engineering, Pohang University of Science and Technology (POSTECH), Pohang, Gyeongbuk, 37673, South Korea
[2] Institute for Convergence Research and Education in Advanced Technology (I-CREATE), Yonsei University, Incheon, 21983, South Korea

* To whom correspondence should be addressed.
Tel: +82 54 279 2144; Email: seungsoo@postech.ac.kr

[†] Joint Authors

Table of Contents

1. Supplementary methods 3

2. Supplementary tables and figures 5

Table S1: Oligonucleotides used in this work 5
Table S2: Distinct nucleic acid specificities of nucleases 7
Table S3: Reported half-lives of nucleic acids relying on various protection methods 8
Figure S1: LOQ values of the MnSOD MB in five different conditions 10
Figure S2: The MG aptamer-specific increase in MG fluorescence 11
Figure S3: Target recognition of DNA aptamers in CDHP 12
Figure S4: Sequence design of hammerhead ribozyme 13
Figure S5: Thermal stabilities of RNA duplexes in CDHP 14
Figure S6: Catalytic yield of hammerhead ribozyme in CDHP 15
Figure S7: Requirements of ribozyme and Mg2+ for substrate cleavage in CDHP 16
Figure S8: Modified oligonucleotides to visualize their nucleolytic degradations 17
Figure S9: Activity recovery of nucleases in diluted CDHP solutions 18
Figure S10: Nuclease activities relying on CDHP concentrations 19
Figure S11: Fraction of intact oligonucleotides at different CDHP concentrations 20
Figure S12: Monitoring of biological degradations of nucleic acids in aqueous condition 21
Figure S13: Monitoring of biological degradations of nucleic acids in CDHP 22
Figure S14: RNase and DNase activity of human saliva 23
Figure S15: Aptameric detection of salivary cortisol in CDHP 24
Figure S16: Structure-switching of ATP aptamer in CDHP 25
Figure S17: Cage effect of CDHP on nucleic acids 26
Figure S18: Cage effect of CDHP on structural compaction of functional nucleic acids 27
Figure S19: Active RNase A even in the presence of excessive RNase inhibitor 28

3. Supplementary references 29

1. Supplementary methods

Target recognition of PS2.M aptamer in CDHP

To investigate the target affinity of PS2.M G-quadruplex aptamer against NMM and thioflavin T in CDHP, 200 mM KCl (10 μl) and 100 mM Tris-HCl (10 μl) (pH 7.6) were mixed with the 4 M CDHP (50 μl) (pH 7.5), 13 μM PS2.M (1 μl), and nuclease-free water (14 μl). After vigorously vortexing, NMM or thioflvain T solution (15 μl) with varying concentrations was added, respectively (final experimental condition: 20 mM KCl, 10 mM Tris-HCl, 2 M CDHP, 130 nM PS2.M, 100 μl volume). The fluorescence intensities of NMM and thioflavin T were recorded at 610 nm (excitation: 399 nm) and 490 nm (excitation: 425 nm), respectively.

Target recognition of St-21-1 in CDHP

The target affinity of St-2-1 was measured by employing streptavidin-coated magnetic beads (Sera-Mag™ Magnetic Streptavidin Microparticles). 4 μl beads were washed twice using 1X PBS buffer, and then washed beads were incubated with 1.14 ml appropriate buffer (1X PBS, 2 M NaCl with 1X PBS, and 2 M CDHP with 1X PBS) for 30 min at room temperature. Thereafter, to the 190 μl bead solution, 10 μl 5'-FAM-labeled St-21-1 aptamer at varying concentrations in appropriate buffer was added. After 90 min incubation at room temperature, the bead-bound aptamer strands were selectively collected using magnet, and they were immediately dissolved in an aqueous buffer (250 mM NaCl and 500 mM Tris-HCl, pH 8.5). The FAM fluoresence was recored at 535 nm (excitation: 485 nm).

Structure-switching of ATP aptamer in CDHP

To prepare the hybridized complex consisting of the ATP aptamer and AD-13 in CDHP, we mixed the 4X ATP binding buffer (25 μl) (1.2 M NaCl, 20 mM $MgCl_2$, and 80 mM Tris-HCl, pH 7.4) with the 4 M CDHP (50 μl), 10 μM ATP aptamer (1 μl), 15 μM AD-13 (1 μl), and nuclease-free water (13 μl). This mixture was heat-treated to hybridize the ATP aptamer and AD-13 (95 °C for 2 min, and cooling down to room temperature at a rate of 0.5°C/s). Finally, an ATP solution (10 μl) with varying concentrations was added to measure a half-maximal effective concentration (EC_{50}) value of the aptamer. Fluorescence signals were recorded with following setting: excitation: 485 nm and emission: 535 nm. In the ATP specificity assay, the concentration of all nucleotide monomers was set to 1 mM.

Aptameric recognition of salivary cortisol in CDHP

Before testing cortisol aptamer-mediated target detection in CDHP, we prepared 10X Cortisol-binding buffer (CBB) (1.37 M NaCl, 50 mM $MgCl_2$, and 0.5 M Tris-HCl, pH 7.4), and then the 10X CBB (10 μl) was mixed with 4 M CDHP (50 μl), nuclease-free water (37.4 μl), 10 μM cortisol aptamer (1 μl), and 10 μM 3'-FAM-labeled signaling strand (1 μl). To the mixture, a cortisol solution (0.5 μl in DMSO) with varying concentrations was added (final experimental condition: 1X CBB, 2 M CDHP, 1 μM cortisol aptamer, 1 μM signaling strand, 0.5% DMSO, 100 μl final volume). Finally, the aptamers in CDHP were incubated for 30 min at room temperature, and then the heat treatment was conducted (95 °C for 3 min,

and cooling to 25 °C with a rate of 0.5 °C/sec). The FAM signal of signaling strand was measured with the following settings: excitation = 485 nm, emission: 535 nm). This experiment was performed in triplicate. In the control experiment with no CDHP, the nuclease-free water instead of 4 M CDHP was used.

To test aptameric recognition of salivary cortisol either in aqueous or CDHP condition, we mixed human saliva (100%), 25 μM cortisol aptamer (0.4 μl), 25 μM 3'-FAM-labeled signaling strand (0.4 μl), and 4 M CDHP (25 μl). To the reaction mixture (50.8 μl), a cortisol solution (0.5 μl) with varying concentrations was added, and subsequently, the volume was decreased down to ~50.5 μl. The prepared sample were incubated at 37 °C for 3,000 min (the control sample with no CDHP was incubated for 30 min. After incubation, the heat treatment and FAM signal measurement were performed as mentioned above.

2. Supplementary tables and figures

Table S1. Oligonucleotides used in this work.

Name	Type	Sequence (5' to 3')	Modification	Source
MnSOD-sensing molecular beacon (MnSOD MB)	DNA	TCAGTTACATTCTCCCAGTTGATT GTAACTGA	5' BHQ2, 3' Cy3	Bioneer
MnSOD mRNA-22	RNA	AAUCAACUGGGAGAAUGUAACU		Bioneer
Malachite green (MG) aptamer	RNA	GGAUCCCGACUGGCGAGAGCCA GGUAACGAAUGGAUCC		Bioneer
Hammerhead ribozyme	RNA	UUGUCACUGAUGAGUCUGAAAAG ACGAAACAUCA		Bioneer
Hammerhead substrate-FAM (Sub)	RNA	UGAUGUCUGACAA	5' FAM	Bioneer
20AU-Forward	RNA	CGAAAAAAAAAAAAAAAAGC		Bioneer
50AU-Forward	RNA	CGGGCAAAAAAAAAACGGGC		Bioneer
80AU-Forward	RNA	CGGGCGCGAAAAGCGCGGGC		Bioneer
20AU-Reverse	RNA	GCUUUUUUUUUUUUUUUUCG		Bioneer
50AU-Reverse	RNA	GCCCGUUUUUUUUUUGCCCG		Bioneer
80AU-Reverse	RNA	GCCCGCGCUUUUCGCGCCCG		Bioneer
Single mismatched RNA	RNA	AAUCAACUGGUAGAAUGUAACU		Bioneer
Double mismatched RNA	RNA	AAUCAACUGGUAGCAUGUAACU		Bioneer
PS2.M	DNA	GTGGGTAGGGCGGGTTGG		IDT
Scrambled MG aptamer	RNA	ACCGUUGCCGGACGAGACUGAU ACGCGGAAGUCGCAAG		Bioneer
St-21-1	DNA	ATTGACCGCTGTGTGACGCAACA CTCAAT	5' FAM	Bioneer

G:C-rich hammerhead ribozyme	RNA	CCCCACUGAUGAGGGGAAACCC GAAACCC		IDT
G:C-rich hammerhead substrate	RNA	GGGUCUGGGG	5' FAM	IDT
Non-substrate (N-Sub)	RNA	GUAUGGUCGUUAG	5' FAM	Bioneer
G-substrate-FAM (G-Sub)	RNA	UGAUGUGUGACAA	5' FAM	Bioneer
MnSOD mRNA-22-Cy5	RNA	AAUCAACUGGGAGAAUGUAACU	3' Cy5	Bioneer
MG aptamer-FAM	RNA	GGAUCCCGACUGGCGAGAGCCA GGUAACGAAUGGAUCC	5' FAM	Bioneer
MnSOD-sensing molecular beacon-Cy3 (MnSOD MB-Cy3)	DNA	TCAGTTACATTCTCCCAGTTGATT GTAACTGA	3' Cy3	Bioneer
G-substrate	RNA	UGAUGUGUGACAA		Bioneer
Cortisol aptamer	DNA	CTC TTG GGA CGA CGC CCG CAT GTT CCA TGG ATA GTC TTG ACT AGT CGT CCC	5’ BHQ1	Bioneer
Signaling strand	DNA	GTC GTC CCA AGA G	3’ FAM	Bioneer
ATP aptamer	DNA	AATTCTGGGGGAGCCTTTTGTGG GTAGGGCGGGTTGGTTTTGCCCC GGAGGAGGAATT	5' FAM, 3' BHQ1	Bioneer
Antisense DNA (AD-13)	DNA	CAACCCGCCCTAC		Bioneer
fret-MG aptamer	RNA	GGAUCCCGXACUGGCGAGAGCC AGGUAACGAYAUGGAUCC	X = Cy3, Y = Cy5	IDT
fret-G-quadruplex	DNA	TAGGGTXTAGGGTTAGGGTYTAGG G	X = Cy3, Y = Cy5	IDT

Table S2. Distinct nucleic acid specificities of seven different nucleases (1–4), and their potential nucleic acid targets used in this work.

Nuclease	Type	Targeted nucleic acid region	Potential targets among our oligonucleotides
**RNase A**	Endonuclease for RNA	All RNAs except for the RNA strand within an RNA:DNA hybrid	MG aptamer, hammerhead ribozyme, and hammerhead ribozyme-substrate complex
**RNase H**	Endonuclease for RNA	The RNA strand within an RNA:DNA hybrid	MnSOD mRNA, hybridized with the molecular beacon
**Exonuclease T (RNase T)**	Exonuclease (3' to 5' direction) for RNA and endonuclease for DNA	Both single-stranded RNAs and DNAs	MG aptamer, molecular beacon, MnSOD mRNA, hammerhead ribozyme, and hammerhead ribozyme-substrate complex
**DNase I**	Endonuclease for DNA	All DNAs	Molecular beacon with or without its target mRNA
**T5 exonuclease**	Exonuclease (5' to 3' direction) for DNA	Linear double-stranded DNAs	Molecular beacon with or without its target mRNA, MG aptamer, hammerhead ribozyme, and hammerhead ribozyme-substrate complex
**Exonuclease V**	Exonuclease (both 3' to 5' and 5' to 3' directions) for DNA	Linear double-stranded DNAs and RNAs	Molecular beacon with or without its target mRNA
**S1 nuclease**	Endonuclease for DNA and RNA	Single-stranded regions of both DNAs and RNAs	MG aptamer, molecular beacon, molecular beacon-MnSOD complex, hammerhead ribozyme, and hammerhead ribozyme-substrate complex

Table S3. Reported half-lives of nucleic acids relying on various protection methods.

Protection method	Type of nucleic acids	Nuclease	Half-life before protection	Half-life after protection	Relative increase in half-life[b]
Conjugation to nanoparticles (5)	DNA	DNase I	23 min	100 min	4.3
Addition of minor groove binder (6)	DNA	10% mouse serum	<3 h	>24 h	>6
Use of close-packed helix structure (7)	DNA	DNase I	<2.5 min	<30 min	>12
UV cross-linking for T-T dimers (8)	DNA	DNase I	<10 min	>60 min	>6
Complexation between HSA[a] and dendritic DNA (9)	DNA	10% fetal bovine serum	33 min	22 h	40
Oligolysine coating (10)	DNA	10% fetal bovine serum	5 min	50 min	10
Cross-linked oligolysine coating (11)	DNA	DNase I	16 min	66 h	~250
Cholesterol conjugation (12)	DNA	rat serum	<1 min	9-11 min	>10
2'-NH_2 or 2'-C-allyl substitutions (13)	RNA	human serum	<0.1 min	5-8 h	~5,000
Combination of 2'-NH_2 or 2'-C-allyl substitutions with 3'-3'-linked caps (13)	RNA	human serum	<0.1 min	>260 h	>100,000
Phosphorothioate linkages (14)	DNA	DNase I	<1 min	~30 min	>30

2’ NH_2 substitutions of pyrimidine (15)	RNA	85% human serum	<10 min	~8 h	>48
Terminal ligation (16)	DNA	human plasma	16 h	50 h	3.1
End blocks by locked nucleic acids (17)	DNA	human serum	1.5 h	15 h	10

[a]Human serum albumin. [b]The relative increase in half-life was calculated by dividing the half-life of the protected oligonucleotide by that of unprotected one.

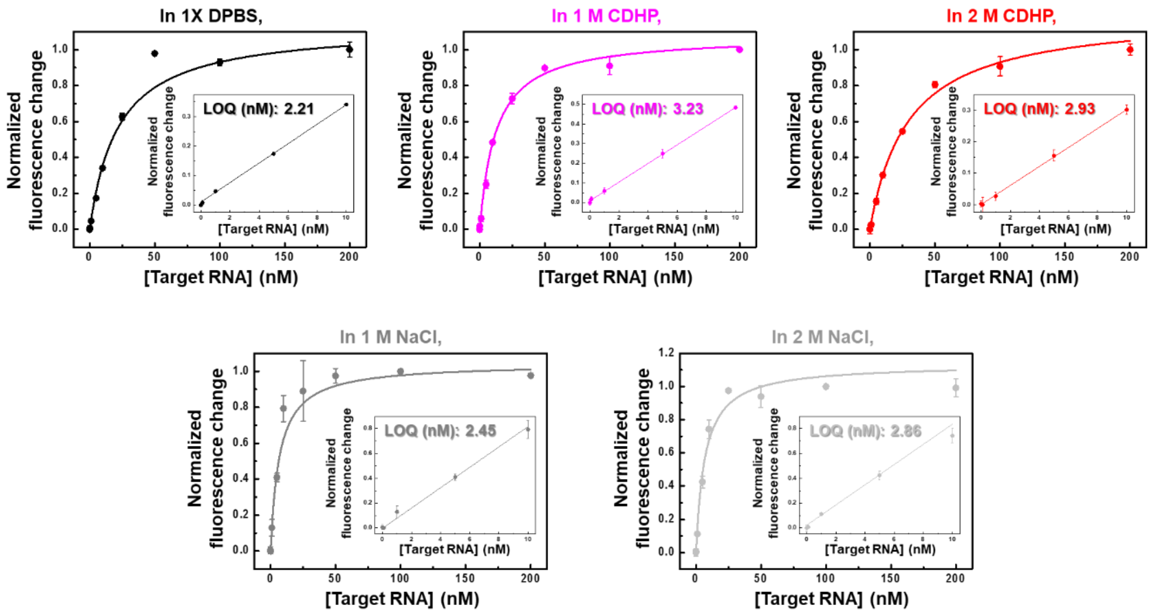

Figure S1. LOQ values of the MnSOD MB in five different conditions: 1X DPBS, 1 and 2 M CDHP (with 1X DPBS), and 1 and 2 M NaCl (with 1X DPBS). When we calculated LOQ value of MnSOD MB in five different conditions based on Cy3 signal (575 nm), we found that the target sensitivity of MnSOD MB is almost unchanged irrespective of cation and anion types and their concentrations. In the range of MnSOD mRNA-22 concentrations from 0.1 to 10 nM, the fluorescence intensities are linear (inset), yielding the LOQ value based on 10σ/slope method (σ is the standard deviation of the blank signal).

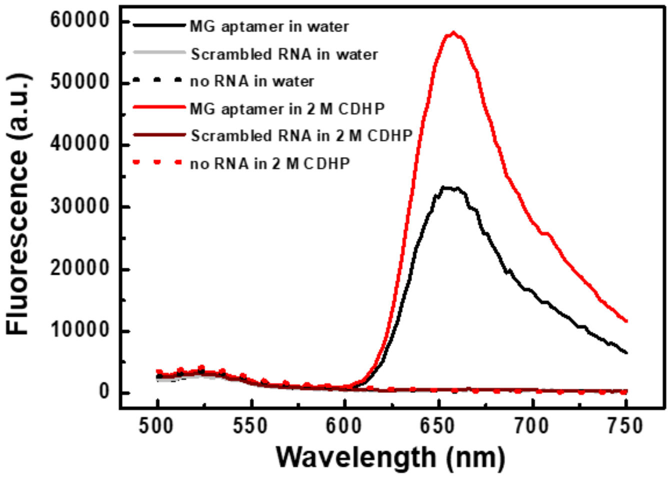

Figure S2. The aptamer-specific increase in fluorescent signal of MG in both water and 2 M CDHP. The scrambled MG aptamer (100 nM) fails to recognize the MG, leading no increase in the MG fluorescence. In this experiment, the fluorescence emission profile was recorded in a 500-750 nm wavelength range with emission bandwidth: 1 nm (excitation setting: 450 nm). Scrambled RNA sequence was designed by using the bioinformatics tools of GenScript (https://www.genscript.com/tools/create-scrambled-sequence).

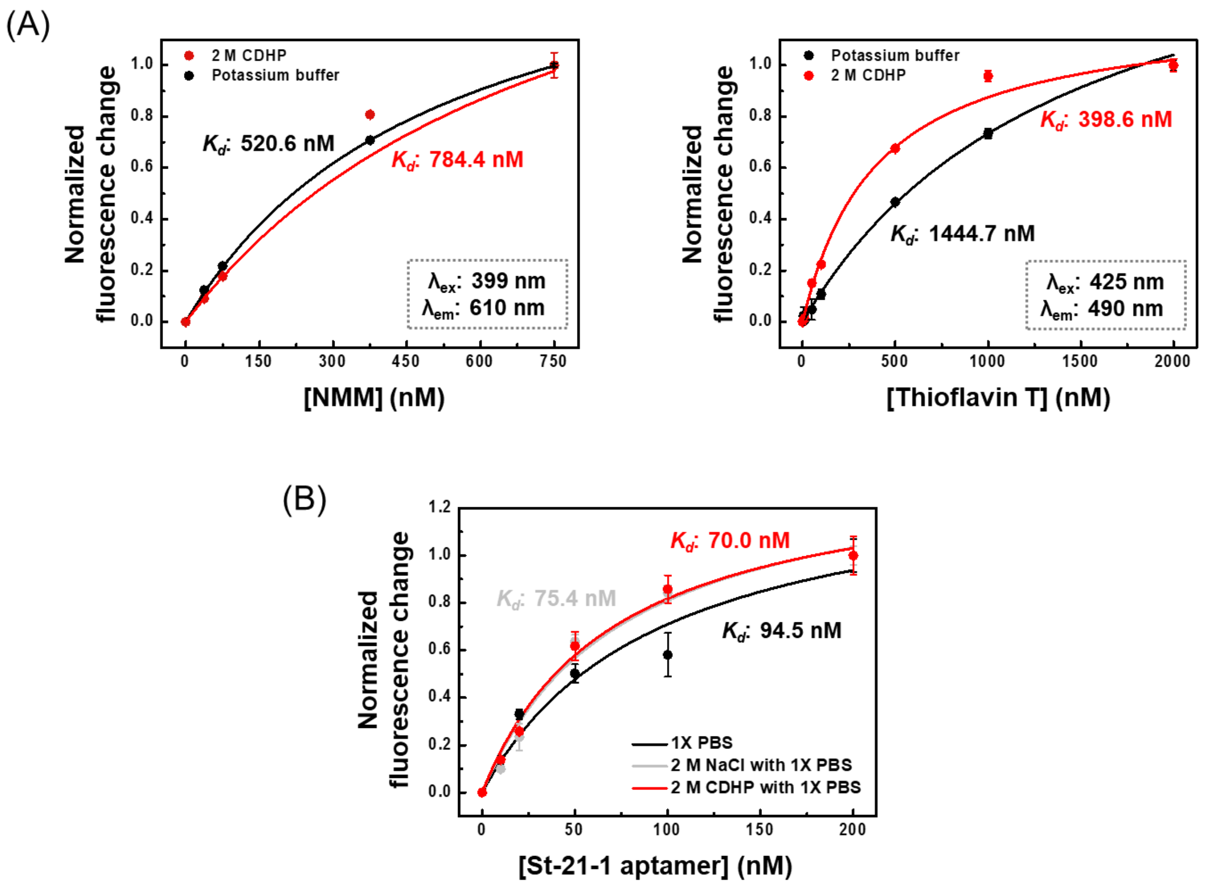

Figure S3. Successful target recognition of DNA aptamers in CDHP and in optimal buffer. (A) The small molecule recognition of the PS2.M aptamer. The PS2.M in 2 M CDHP successfully bind to its two cognate targets (NMM and thioflavin T) like that in the aqueous condition (100 mM KCl, 10 mM Tris-HCl, pH 7.6). When the target affinity (K_d) of PS2.M is investigated, the CDHP solvation (2 M CDHP, 100 mM KCl, 10 mM Tric-HCl, pH 7.6) slightly increases the K_d value of PS2.M to NMM (left), but decreases that of PS2.M to thioflavin T (right). (B) The large protein recognition of the St-21-1 aptamer. When the St-21-1 recognizes the streptavidin protein in 1X PBS, it shows K_d value of 94.5 nM. However, high ionic strengths of both 2 M NaCl and 2 M CDHP lower K_d values down to 75.4 and 70.0 nM, respectively.

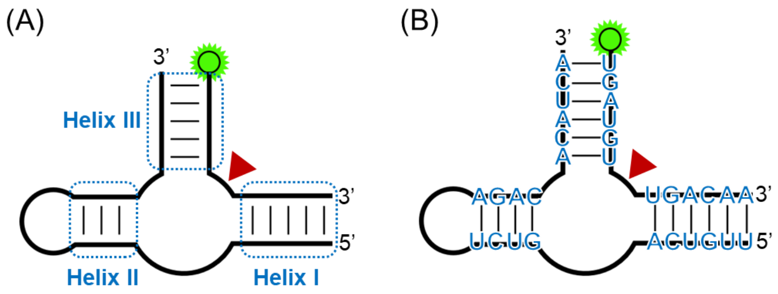

Figure S4. Engineering of hammerhead ribozyme sequence. (A) Three stem formations (blue dotted box) when the hammerhead ribozyme hybridizes with its substrate. (B) The structure of engineered hammerhead ribozyme. To increase the thermal stabilities of stem regions in the CDHP, we intentionally inserts a few A:U pairs in all three stems.

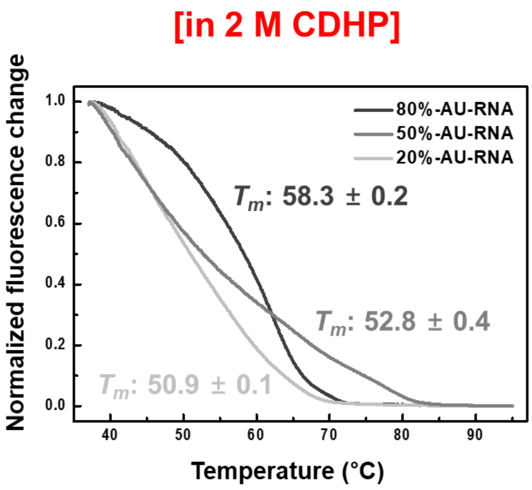

Figure S5. Thermal stabilities of RNA duplexes in CDHP. In 2 M CDHP solution, the RNA duplex with higher A-U content is more thermodynamically stable than that with lower A-U content. For instance, the melting temperature (T_m) of RNA duplex with 80% A-U content (80AU-RNA) is 7.4 °C higher than that with 20% A-U content (20AU-RNA). In this assay, EvaGreen® dye that can intercalate to an RNA duplex was exploited to measure T_m (18). The concentration of all RNA oligonucleotides was set to 2 μM. To obtain fluorescence melting curves, the fluorescent signals of 18 μl solution was measured by LightCycler® 96 (Roche). Fluorescent changes of EvaGreen® dye were recorded within 37-98 °C temperature range with 0.1 °C increments.

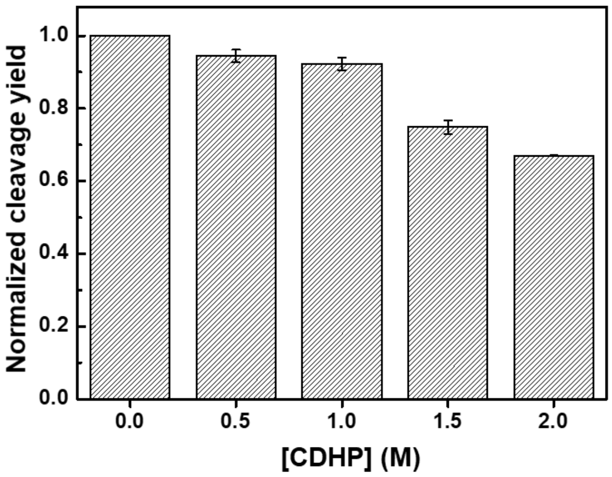

Figure S6. Normalized cleavage yield of hammerhead ribozyme relying on CDHP concentrations. In CDHP, the engineered hammerhead ribozyme shows a diminished catalytic yield compared to that in aqueous solution. Based on Figure 2F, we obtained the band intensities of substrates and cleaved products by using GelQuant.Net software provided by biochemlabsolutions.com. For the normalization, we calculated the catalytic yield of hammerhead ribozyme without CDHP as one.

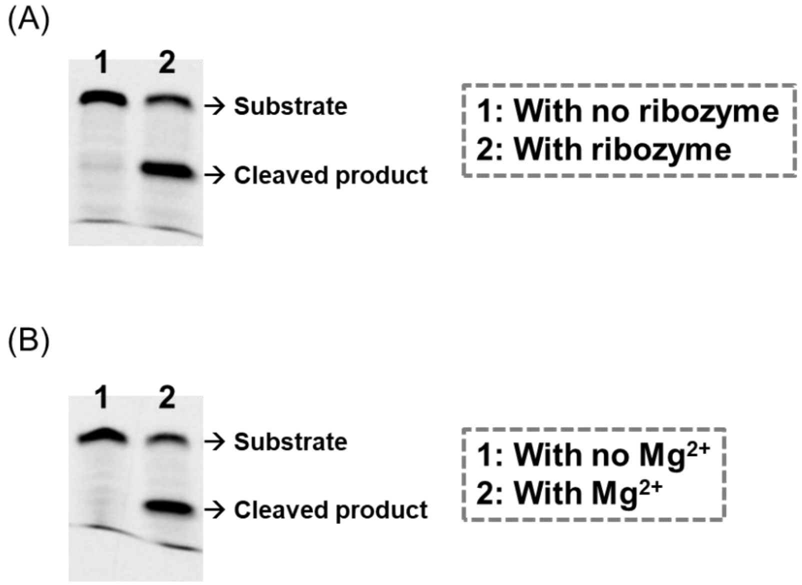

Figure S7. Requirement of both ribozyme and Mg^{2+} for the successful ribozymatic cleavage in 2 M CDHP. (A) The cleaved product is only detectable in the presence of the ribozyme. (B) Even with the ribozyme, to catalyze the RNA-mediated substrate cleavage, Mg^{2+} is also required.

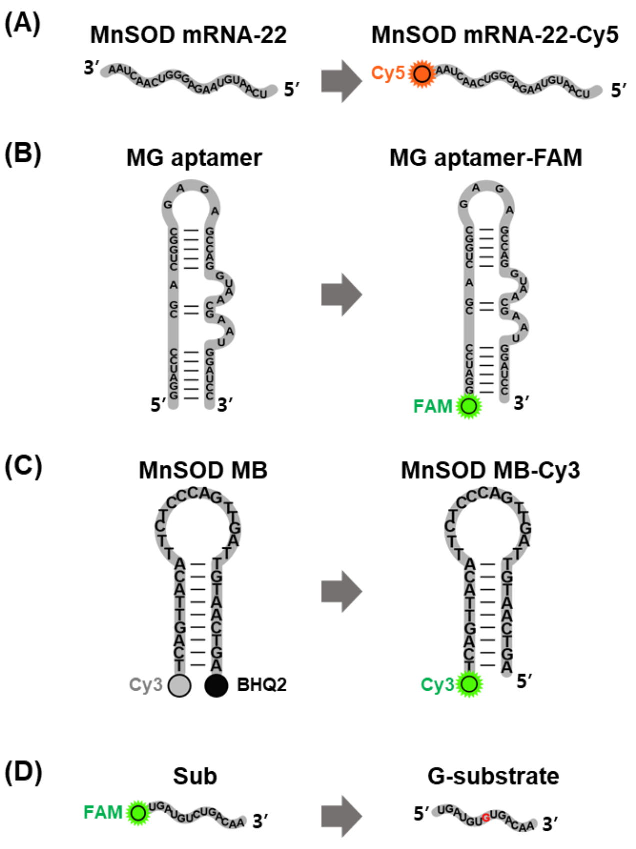

Figure S8. Partly modified oligonucleotides to easily visualize nuclease-mediated degradations of FNAs in gel electrophoresis. (A) Cy5 incorporation at the 5’ end of MnSOD mRNA-22. (B) 5’-FAM labeling of MG aptamer. (C) Removal of 5’-labeled-BHQ2 of MnSOD MB. (D) The use of G-substrate instead of Sub to exclude a ribozymatic cleavage when investigating nuclease activities in water and CDHP.

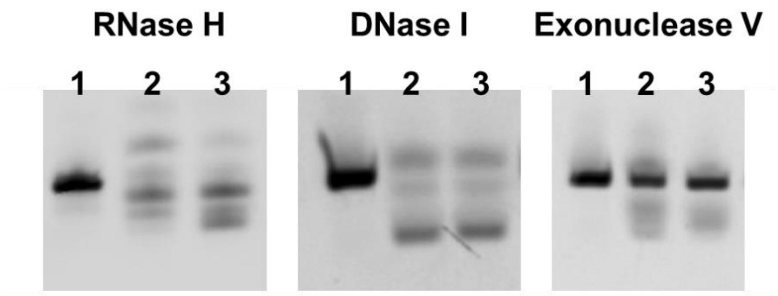

Lane 1: no nuclease
Lane 2: Nuclease-containing aqueous buffer, diluted with water (20x dilution)
Lane 3: Nuclease-containing CDHP (2 M), diluted with water (20x dilution)

Figure S9. Activity recovery of various nucleases that were incubated in 2 M CDHP. We mixed a nuclease either with aqueous buffer or with 2 M CDHP at 37 °C (for 30 min), and then diluted nuclease-containing solutions with excess water (20x dilution). When these diluted nucleases were incubated with target nucleic acids, all the analyzed nucleases successfully exhibit nucleolytic activities, proving that the CDHP environment does not denature protein scaffolds, but inhibits the hydrolytic reaction.

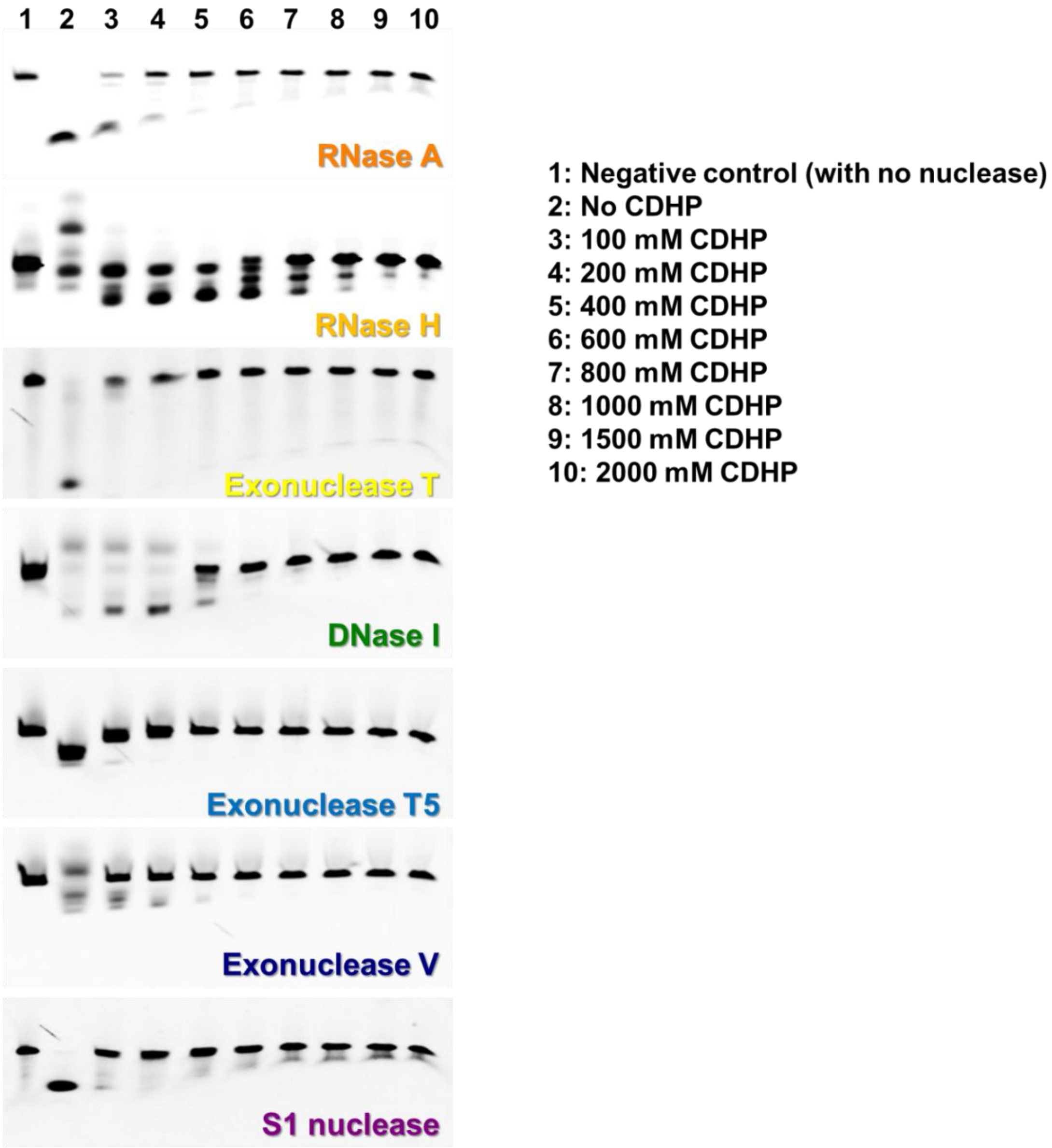

Figure S10. CDHP concentration-dependent hydrolysis activities of seven different nucleases. All the analyzed nucleases are effectively inhibited in a CDHP concentration-dependent manner (incubation for 30 min at 37 °C). Exonuclease T5 and S1 nuclease are readily inhibited by the presence of CDHP (~200-400 mM), whereas RNase H is most resistant to the CDHP environment among seven nucleases, so >1.5 M CDHP is required to completely block its hydrolytic action. In this experiments, to investigate the nuclease activities of RNase A, exonuclease T, and S1 nuclease, MG aptamer-FAM was employed, and MnSOD MB-Cy3 was used as a target nucleic acid for DNase I, exonuclease T5, and exonuclease V. In the case of RNase H assay, we analyzed fluorescent signals of MnSOD mRNA-22-Cy5, hybridized with MnSOD MB-Cy3.

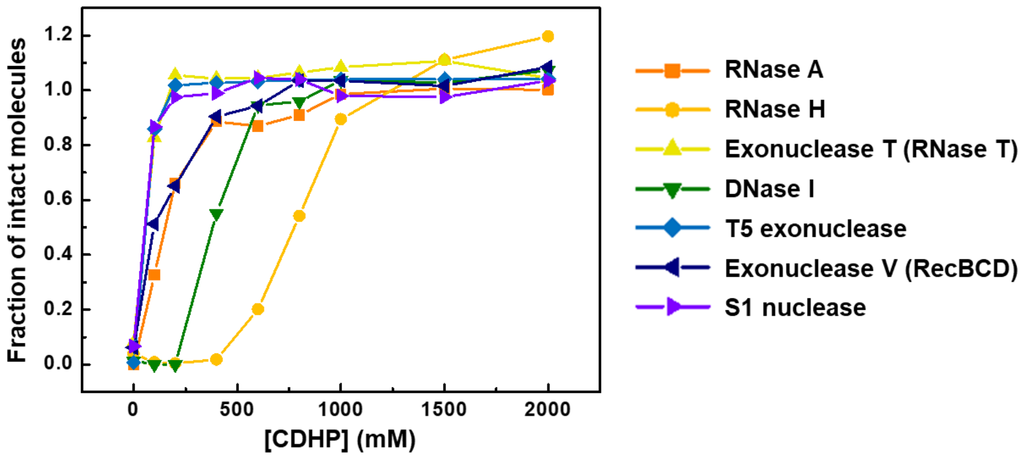

Figure S11. Fraction of intact oligonucleotides at different CDHP concentrations (0-2000 mM) in the presence of each nuclease. Based on Figure S9, the fraction of intact molecule was calculated by dividing the fluorescent signal of an intact oligonucleotide by that of the oligonucleotide with no nuclease (Figure S10, negative control). To investigate gel band intensities, we used GelQuant.Net software provided by biochemlabsolutions.com.

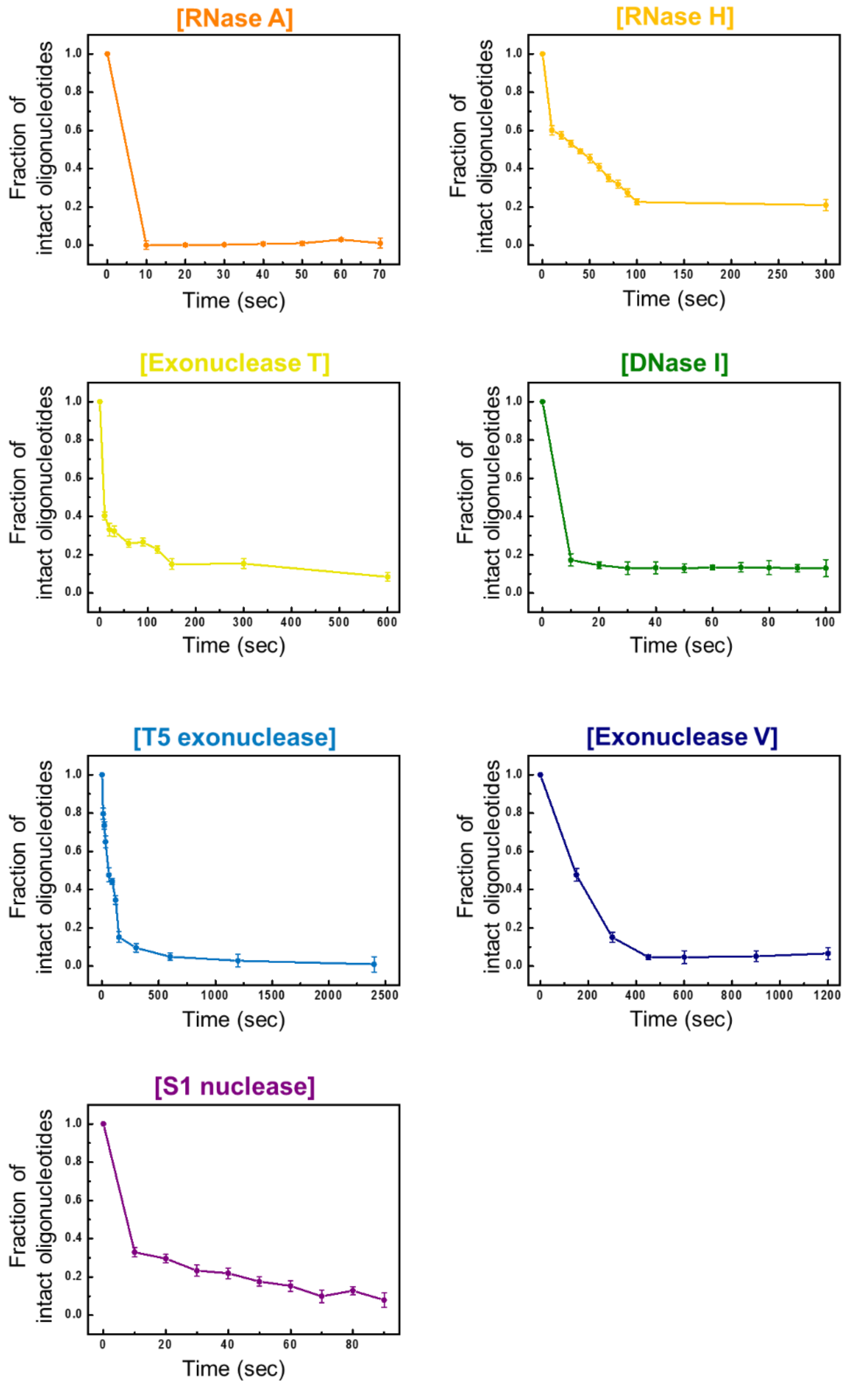

Figure S12. Monitoring time-dependent oligonucleotide degradations in the presence of nucleases in the aqueous condition. The MG aptamer-FAM was employed for RNase A, exonuclease T, and S1 nuclease experiments, and the MnSOD MB-Cy3 was used for DNase I, T5 exonuclease, and exonuclease V assays. Moreover, the MnSOD mRNA-22-Cy5, hybridized with MnSOD MB-Cy3, was analyzed for the RNase H experiment.

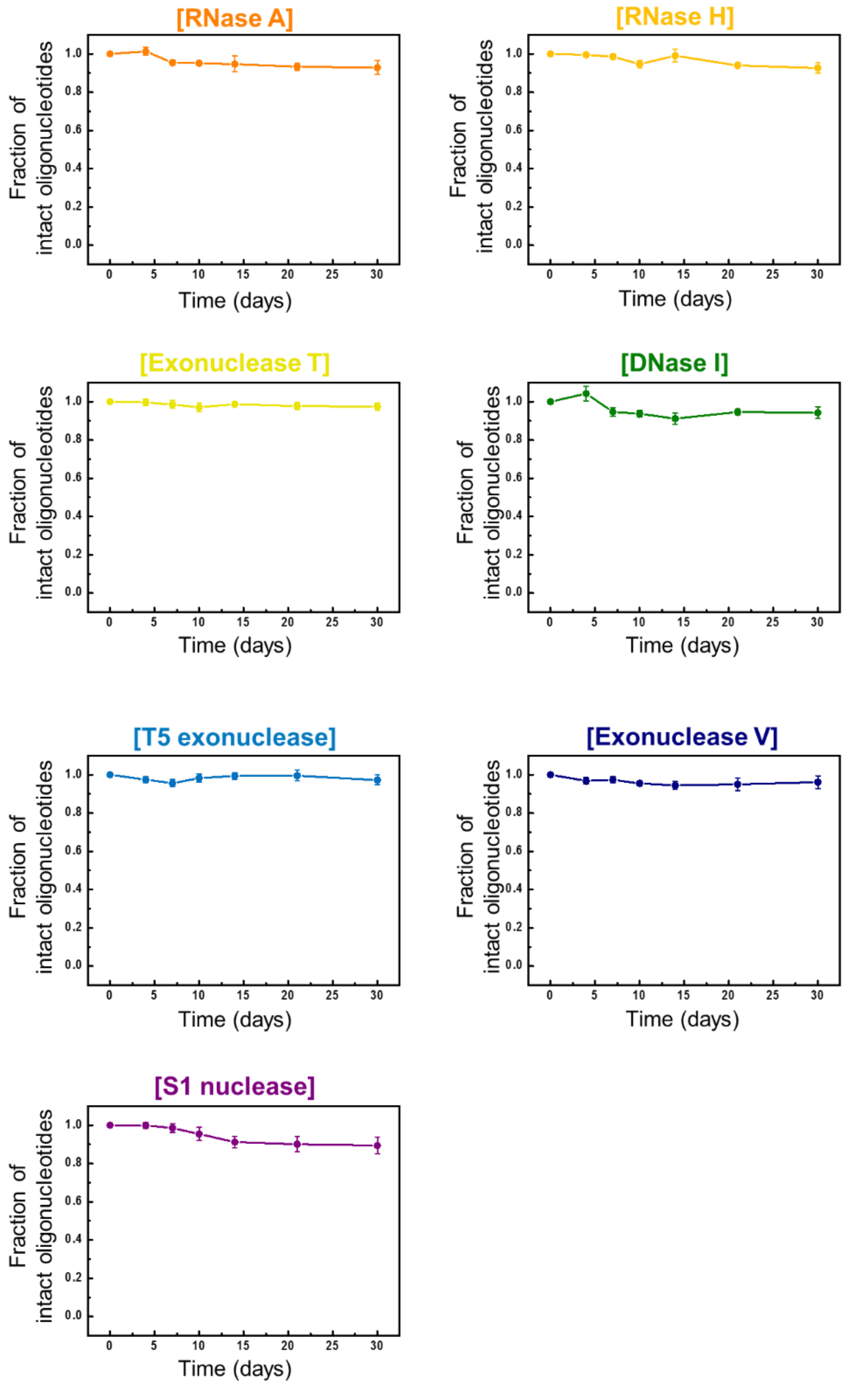

Figure S13. Monitoring time-dependent oligonucleotide degradations in the presence of nucleases in 2 M CDHP. For 1-month incubation at 37 °C, only negligible nuclease-driven degradations are observed.

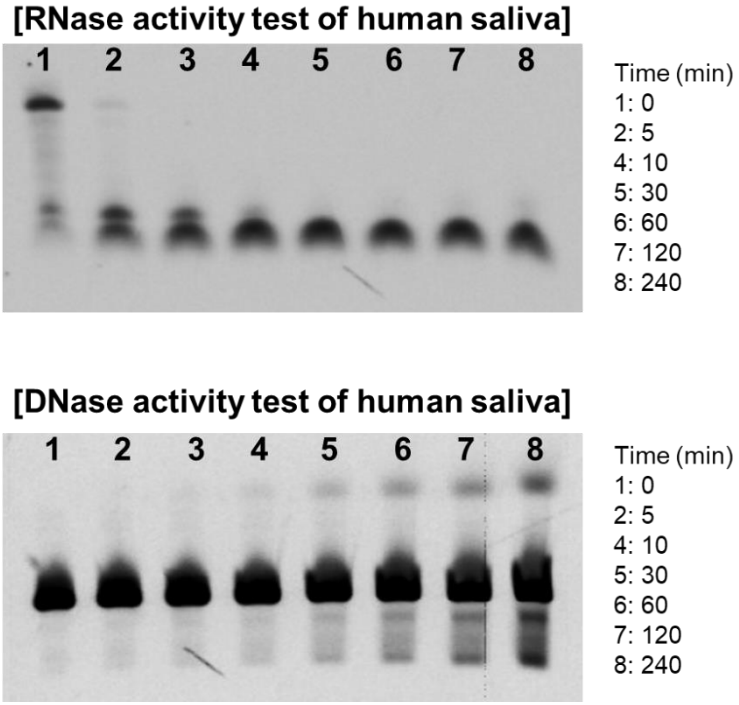

Figure S14. RNase and DNase activities of human saliva (50%). The non-treated saliva shows the high RNase activity, completely digesting 2.5 μM MG aptamer-FAM within 10 min at 37 °C (total volume: 10 μl) (top). However, as the DNase activity of saliva is quite lower than the RNase activity, most of MnSOD MB-Cy3 (2.5 μM) remains intact even after 4 h incubation (bottom).

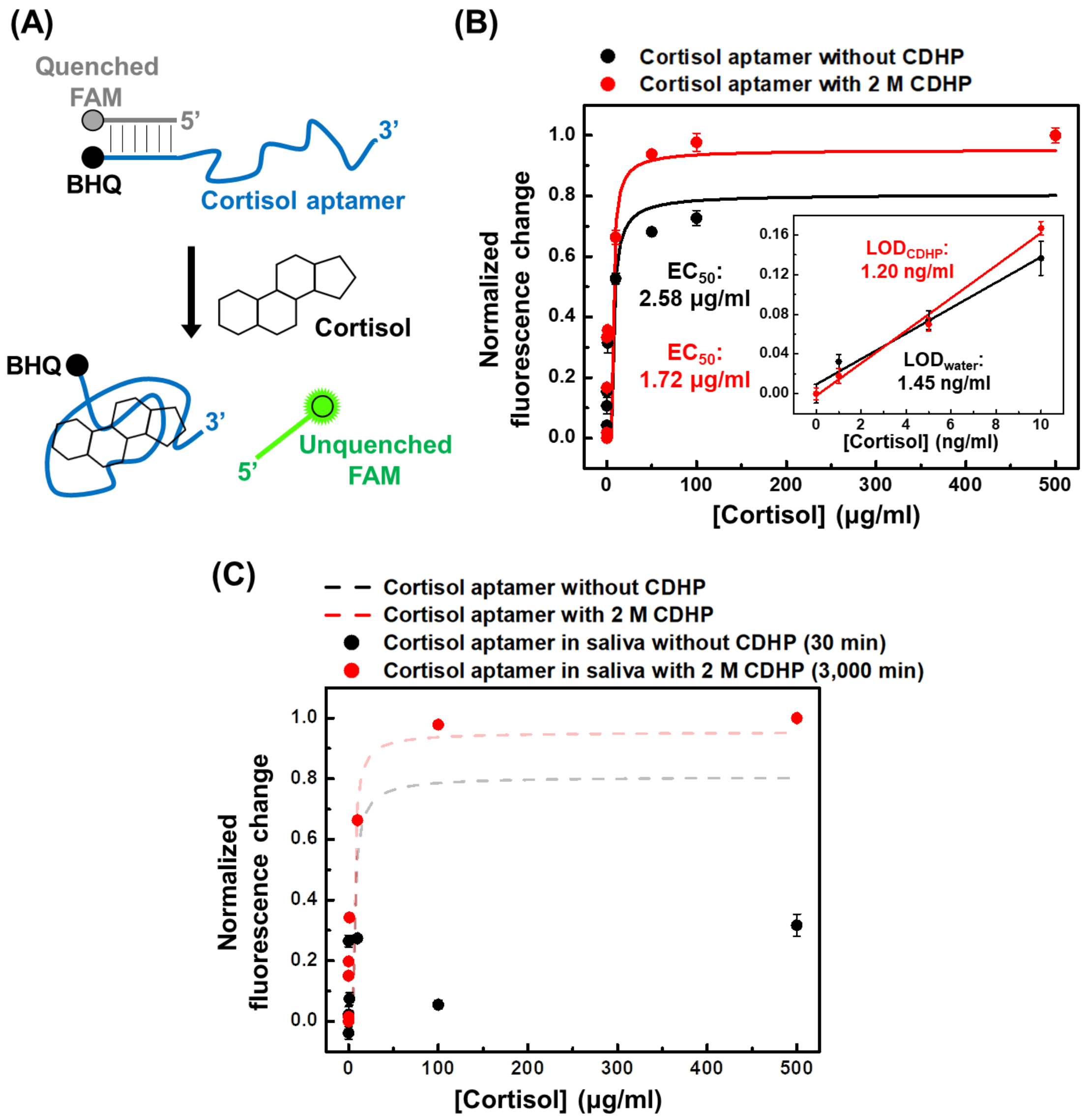

Figure S15. Aptameric detection of salivary cortisol in CDHP. (A) Rational design of cortisol-sensing aptasensor consisting of a 5'-BHQ1-labeled cortisol aptamer and a 3'-FAM-labeled signaling strand. When the aptamer recognizes its cognate target, the signaling strand can be released, thereby producing the FAM fluorescence signals in a cortisol concentration-dependent manner. (B) The aptamer-mediated cortisol detection in 2 M CDHP (with 1X CBB) and the aqueous condition (1X CBB only). In both conditions, the cortisol aptamer successfully recognize its cognate target. Notably, in the range of cortisol concentrations up to 10 ng/ml, fluorescent signals are measured to be linear (inset). The LOD values are calculated by 3σ/slope method (σ is the standard deviation of blank signal). (C) Salivary cortisol detection of aptamers with 2 M CDHP. Without CDHP, cortisol concentration-irrelevant fluorescent signals of aptasensor were observed in saliva (30 min incubation). However, with 2 M CDHP, the cortisol aptamers successfully bind to its target with no hydrolysis even after 3,000 min incubation at 37 °C, evidenced by desired FAM signals in a cortisol concentration-dependent manner like the fully intact aptamers without saliva.

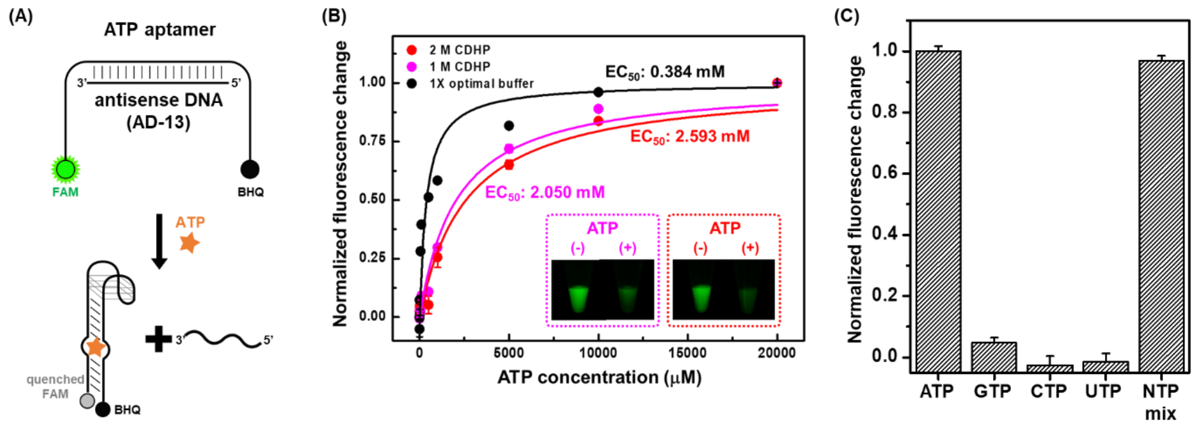

Figure S16. Successful structure-switching of ATP aptamer either in CDHP (1 and 2 M) or in 1X optimal buffer. (A) ATP-induced structure-switching of ATP aptamer. The ATP aptamer in which central domain is hybridized with antisense DNA (AD-13) can undergo ATP-specific conformational change, releasing AD-13 and subsequently inducing close proximity between 5' FAM and 3' BHQ1 for decreased fluorescent signals(19, 20). (B) Fluorescent signal changes of ATP aptamer in an ATP concentration-dependent manner. In both 1 and 2 M CDHP, the ATP aptamer successfully performs its pre-defined structure-switching, decreasing FAM fluorescence (inset). Relying on ATP concentration-dependent fluorescent signals, a half-maximal effective concentration (EC_{50}) of ATP aptamer is obtained in three different conditions: 1X optimal buffer, 1 and 2 M CDHP (with 1X optimal buffer). 1X optimal buffer consists of 300 mM NaCl, 5 mM $MgCl_2$, 20 mM Tris-HCl (pH 7.4). (C) ATP-specific and selective structure-switching of ATP aptamer in 2 M CDHP. Even in 2 M CDHP, the ATP aptamer retains its specificity and selectivity to the ATP molecule, with no response to other nucleotides (GTP, CTP and UTP). In this assay, the concentration of all nucleotide monomers was set to 1 mM.

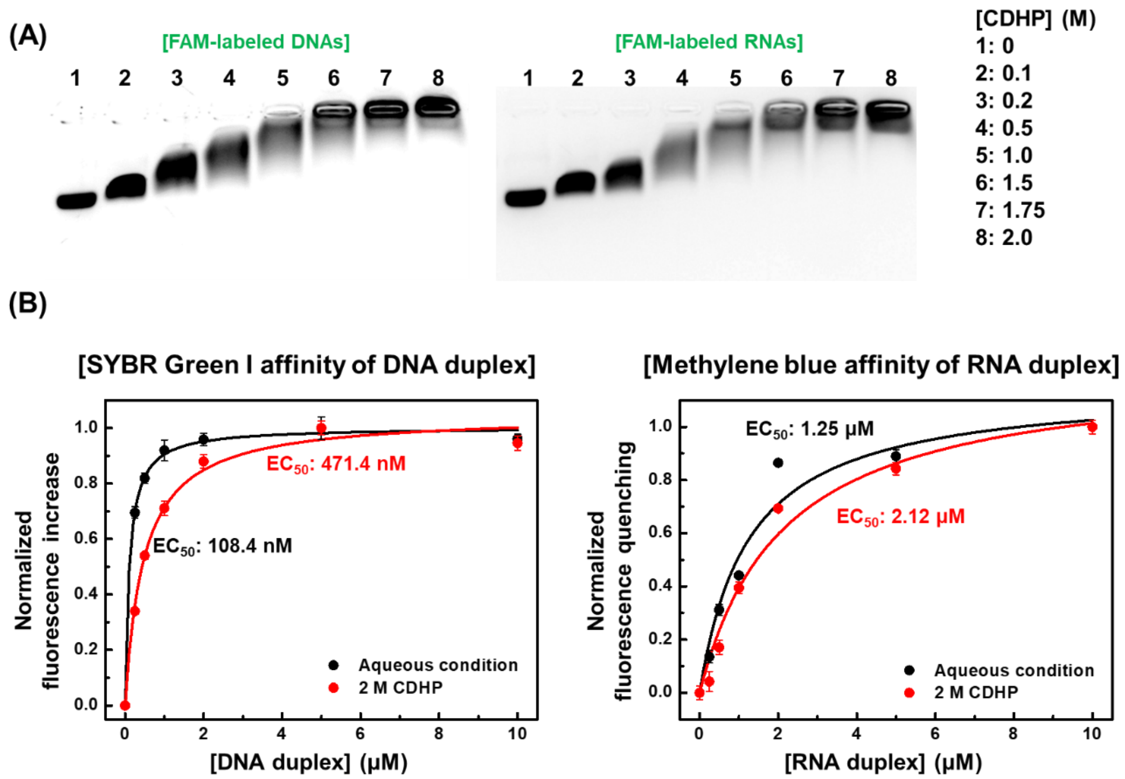

Figure S17. Cage effect of CDHP on nucleic acids. (A) Charge neutralization of oligonucleotides relying on CDHP concentrations. At the high CDHP concentration (2 M), the migration of both FAM-labeled DNAs and RNAs was fully retarded in agarose gel electrophoresis as their negatively-charged phosphate backbones were neutralized by choline cations. Experimental condition: 1X TBE-1.2% agarose gel (100V and 12 min) and 1 μM oligonucleotides. (B) The binding affinity of minor groove-binding fluorescent dyes to nucleic acid duplexes with or without CDHP (2 M). When we investigated EC_{50} for SYBR Green I by titrating the DNA duplex, its binding affinity is decreased in the presence of CDHP that can be complexed with the minor grooves of nucleic acids. Likewise, when titrating RNA duplex, we found that 2 M CDHP condition lowered the binding affinity of methylene blue to RNA duplex.

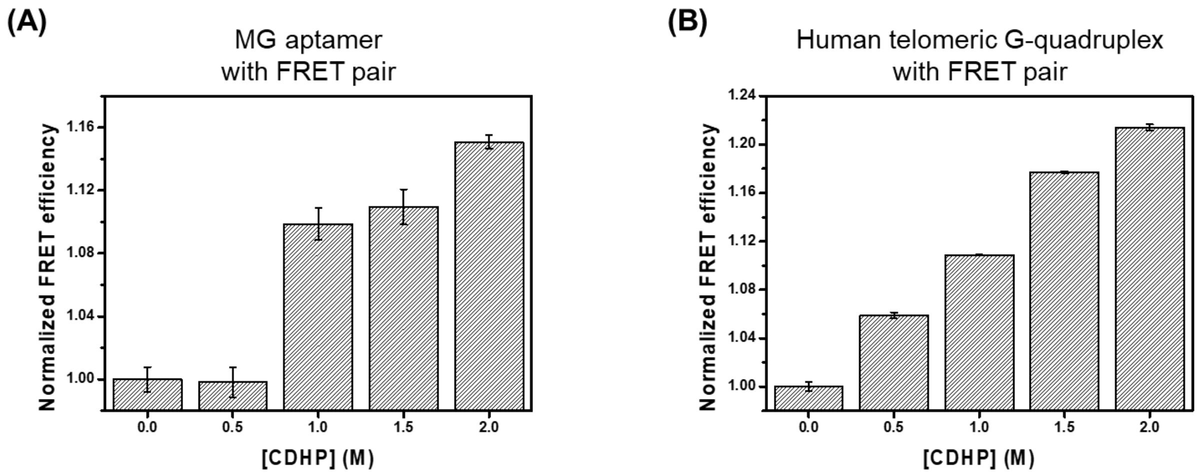

Figure S18. Cage effect of CDHP on structural compaction of functional nucleic acids. To calculate the FRET efficiencies of functional nucleic acids, which are internally labeled with Cy3 and Cy5 fluorophores, at varied CDHP concentrations (up to 2 M), the Cy3 signal (excitation: 530 nm, emission: 575 nm) and Cy5 signal (excitation: 530 nm, emission: 675 nm) were measured. To normalize the FRET efficiency, the FRET efficiency of a sample was divided by that of a sample without CDHP (0.0 M). All samples included 30 mM Tris-HCl (pH 7.5). (A) The normalized FRET efficiency of the FRET pair-labeled MG aptamer at varied CDHP concentrations. In this experiment, fret-MG aptamer was used (Table S1). (B) The normalized FRET efficiency of human telomeric G-quadruplex at varied CDHP concentrations. fret-G-quadruplex was used. As negligible differences in quantum yields of Cy3- and Cy5-labed oligonucleotides at varying CDHP concentrations were observed (data was not shown), we did not consider the quantum yield of fluorophore dyes when calculating the FRET efficiency.

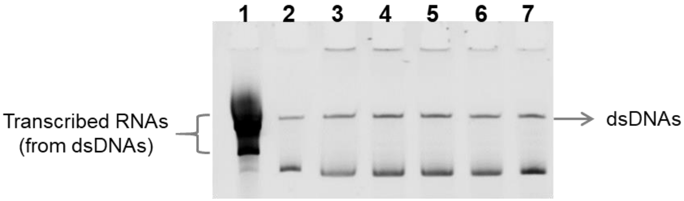

1: control (with no RNase A)
2: RNase A
3: RNase A with RNasin® Ribonuclease Inhibitor (10X)
4: RNase A with RNasin® Ribonuclease Inhibitor (8X)
5: RNase A with RNasin® Ribonuclease Inhibitor (6X)
6: RNase A with RNasin® Ribonuclease Inhibitor (4X)
7: RNase A with RNasin® Ribonuclease Inhibitor (2X)

Figure S19. Catalytically active RNase A even in the presence of excessive RNase inhibitors. When *in vitro* transcribed RNAs from 165-nt long dsDNA with T7 promoter (lane 1) was incubated with RNase A (lane 2), RNase A fully digested RNA molecules as expected. To preserve transcribed RNAs against RNase A, the excess amounts of commercially available RNase inhibitor (RNasin® Ribonuclease Inhibitor from Promega) were employed (lane 3-7), but nucleases still effectively degraded RNAs, and only dsDNA molecules remained. Incubation for 30 min at 37 °C. Nucleic acids were stained by SYBR Gold I.

3. Supplementary references

1. Garrett, R.H. and Grisham, C.M. (2016) Biochemistry Cengage Learning.
2. Blumberg, D.D. (1987) Creating a ribonuclease-free environment. *Methods Enzymol.*, **152**, 20–24.
3. Yang, W. (2011) Nucleases: diversity of structure, function and mechanism. *Q. Rev. Biophys.*, **44**, 1–93.
4. Farrell Jr, R.E. (2009) RNA Methodologies: laboratory guide for isolation and characterization Academic Press.
5. Seferos, D.S., Prigodich, A.E., Giljohann, D.A., Patel, P.C. and Mirkin, C.A. (2009) Polyvalent DNA nanoparticle conjugates stabilize nucleic acids. *Nano Lett.*, **9**, 308–311.
6. Wamhoff, E.C., Romanov, A., Huang, H., Read, B.J., Ginsburg, E., Knappe, G.A., Kim, H.M., Farrell, N.P., Irvine, D.J. and Bathe, M. (2022) Controlling Nuclease Degradation of Wireframe DNA Origami with Minor Groove Binders. *ACS Nano.*, **16**, 8954–8966.
7. Castro, C.E., Kilchherr, F., Kim, D.N., Shiao, E.L., Wauer, T., Wortmann, P., Bathe, M. and Dietz, H. (2011) A primer to scaffolded DNA origami. *Nat. Methods*, **8**, 221–229.
8. Gerling, T., Kube, M., Kick, B. and Dietz, H. (2018) Sequence-programmable covalent bonding of designed DNA assemblies. *Sci. Adv.*, **4**, eaau1157.
9. Lacroix, A., Edwardson, T.G.W., Hancock, M.A., Dore, M.D. and Sleiman, H.F. (2017) Development of DNA Nanostructures for High-Affinity Binding to Human Serum Albumin. *J. Am. Chem. Soc.*, **139**, 7355–7362.
10. Ponnuswamy, N., Bastings, M.M.C., Nathwani, B., Ryu, J.H., Chou, L.Y.T., Vinther, M., Li, W.A., Anastassacos, F.M., Mooney, D.J. and Shih, W.M. (2017) Oligolysine-based coating protects DNA nanostructures from low-salt denaturation and nuclease degradation. *Nat. Commun.*, **8**, 1–9.
11. Anastassacos, F.M., Zhao, Z., Zeng, Y. and Shih, W.M. (2020) Glutaraldehyde Cross-Linking of Oligolysines Coating DNA Origami Greatly Reduces Susceptibility to Nuclease Degradation. *J. Am. Chem. Soc.*, **142**, 3311–3315.
12. Lee, C.H., Lee, S.H., Kim, J.H., Noh, Y.H., Noh, G.J. and Lee, S.W. (2015) Pharmacokinetics of a Cholesterol-conjugated Aptamer Against the Hepatitis C Virus (HCV) NS5B Protein. *Mol. Ther. - Nucleic Acids*, **4**, e254.
13. Beigelman, L., McSwiggen, J.A., Draper, K.G., Gonzalez, C., Jensen, K., Karpeisky, A.M., Modak, A.S., Matulic-Adamic, J., DiRenzo, A.B., Haeberli, P., *et al.* (1995) Chemical modification of hammerhead ribozymes. Catalytic activity and nuclease resistance. *J. Biol. Chem.*, **270**, 25702–25708.
14. Latimer, L.J.P., Hampel, K. and Lee, J.S. (1989) Synthetic repeating sequence DNAs containing plsosphorothioates: nuclease sensitivity and triplex formation. *Nucleic Acids Res.*, **17**, 1549–1561.
15. Yan, X., Gao, X. and Zhang, Z. (2004) Isolation and Characterization of 2′-amino-modified RNA Aptamers for Human TNFα. *Genom. Proteom. Bioinform.*, **2**, 32–42.
16. Di Giusto, D.A., Knox, S.M., Lai, Y., Tyrelle, G.D., Aung, M.T. and King, G.C. (2006) Multitasking by Multivalent Circular DNA Aptamers. *ChemBioChem*, **7**, 535–544.
17. Kurreck, J., Wyszko, E., Gillen, C. and Erdmann, V.A. (2002) Design of antisense oligonucleotides stabilized by locked nucleic acids. *Nucleic Acids Res.*, **30**, 1911–1918.
18. Wang, J., Pan, X. and Liang, X. (2016) Assessment for Melting Temperature Measurement of Nucleic Acid by HRM. *J. Anal. Methods Chem.*, **2016**.
19. Oh, S.S., Plakos, K., Xiao, Y., Eisenstein, M. and Soh, H.T. (2013) In vitro selection of shape-changing DNA nanostructures capable of binding-induced cargo release. *ACS Nano.*, **7**, 9675–9683.
20. Kang, B., Park, S. V., Soh, H.T. and Oh, S.S. (2019) A Dual-Sensing DNA Nanostructure with an Ultrabroad Detection Range. *ACS Sensors.*, **4**, 2802–2808.
